# In Arabidopsis hybrids and Hybrid Mimics, up‐regulation of cell wall biogenesis is associated with the increased plant size

**DOI:** 10.1002/pld3.174

**Published:** 2019-11-06

**Authors:** Li Wang, Li Min Wu, Ian K. Greaves, Elizabeth S. Dennis, William James Peacock

**Affiliations:** ^1^ Faculty of Science University of Technology Sydney NSW Australia; ^2^ Agriculture and Food Commonwealth Scientific Industrial Research Organisation Canberra ACT Australia

**Keywords:** biomass, defense, heterosis, intercross, transcriptome

## Abstract

Hybrid breeding is of economic importance in agriculture for increasing yield, yet the basis of heterosis is not well understood. In Arabidopsis, crosses between different accessions produce hybrids with different levels of heterosis relative to parental phenotypes in biomass. In all hybrids, the advantage of the F1 hybrid in both phenotypic uniformity and yield gain is lost in the heterogeneous F2. F5/F6 Hybrid Mimics generated from a cross between C24 and Landsberg *erecta* (L*er*) ecotypes demonstrated that the large plant phenotype of the F1 hybrids can be stabilized. Hybrid Mimic selection was applied to Wassilewskija (Ws)/L*er* and Col/L*er* hybrids. The two hybrids show different levels of heterosis. The Col/L*er* hybrid generated F7 Hybrid Mimics with rosette diameter and fresh weight equivalent to the F1 hybrid at 30 DAS; F7 Ws/L*er* Hybrid Mimics outperformed the F1 hybrid in both the rosette size and biomass. Transcriptome analysis revealed up‐regulation of cell wall biosynthesis, and cell wall expansion genes could be a common pathway in increased size in the Arabidopsis hybrids and Hybrid Mimics. Intercross of two independent Hybrid Mimic lines can further increase the biomass gain. Our results encourage the use of Hybrid Mimics for breeding and for investigating the molecular basis of heterosis.

## 1 INTRODUCTION

Hybrids have proved to have great value in agriculture as they produce large gains in biomass and seed yield in a number of crops (Cheng, Zhuang, Fan, Du, & Cao, 2007; Crow, 1998; Dan et al., 2014). The gain in the hybrid plants compared with the performance of parents is referred to as hybrid vigor or heterosis. In Arabidopsis (Fujimoto, Taylor, Shirasawa, Peacock, & Dennis, 2012; Groszmann et al., 2014), maize (Birchler, Auger, & Riddle, 2003; Li, Yang, et al., 2017), and Chinese cabbage (Saeki et al., 2016), hybrid plants have an architecture with larger leaves and the plants are taller compared with the parents. The increased leaf size is due to increased number and size of leaf cells (Fujimoto et al., 2012; Groszmann et al., 2014).

In Arabidopsis, different hybrids differ in growth pattern and level of heterosis, suggesting multiple genetic routes for hybrid vigor. Heterosis is generated through interactions between the two parental genomes and epigenomes in the nucleus of the hybrid. Transcriptome studies revealed that salicylic acid (SA)‐mediated down‐regulation of defense pathway genes contribute to the heterotic phenotype of the C24/L*er* hybrid through increased expression of growth‐promoting genes (Groszmann et al., 2015). In C24/L*er* hybrids, up‐regulation of the transcription factor PHYTOCHROME INTERACTING FACTOR 4 (PIF4) results in increased auxin biosynthesis which promotes plant growth by targeting downstream cell expansion and division genes (Wang et al., 2017). A decreased level of ethylene and its effect in delaying of senescence can also contribute to the extra growth in the hybrid plants (Gonzalez‐Bayon et al., 2019; Song et al., 2018).

Hybrids are an end point to self‐fertilization breeding because of the genomic heterogeneity in the F2 and subsequent generations (Greaves et al., 2015). This provides a challenge to the hybrid industry of how to extend the hybrid advantage beyond the F1. In 1971, Busch, Lucken, and Frohberg, (1971) reported that in wheat pure breeding F5 lines derived from the hybrid plants are equivalent to the F1 hybrid in performance. Similar observations were reported in pea and tomato (Sarawat, Stoddard, & Marshall, 1994; Williams, 1959). No molecular studies were explored beyond these observations. In Arabidopsis, crosses between the C24 and Landsberg *erecta* (L*er*) ecotypes produce hybrids with performance superior to the parental lines in biomass and seed yield (Groszmann et al., 2014; Wang, Liu, et al., 2018). We selfed F2 individuals and coupled these crosses with selection based on the phenotype of the F1 hybrid. These procedures, repeated in successive generations gave, in the F6 and later generations, lines with rosette diameter, biomass, and seed yield comparable to the F1 hybrid (Wang et al., 2015, 2017). Because of genome homozygosity (Wang et al., 2015, 2017), the lines maintained the high yielding phenotype in successive generations. The interactions of the two parental genomes in the F1 hybrid set the level of hybrid vigor that could be achieved by the component alleles of the parents through levels of gene expression and interactions between sequences in the two parental genomes.

These Hybrid Mimics overcame the F1/F2 hurdle, providing a seed source for high yielding crops based on kept‐seed planting.

None of the C24/L*er* Hybrid Mimics outperformed the F1 hybrids (Wang et al., 2017), suggesting that heterosis in these F1 hybrids is the maximum level of vigor which can be achieved by gene interaction between the two parent genomes. To investigate whether Hybrid Mimics can be selected from other Arabidopsis hybrid combinations and to understand the molecular basis of increased plant size, we selected Hybrid Mimics from two hybrid systems involving other ecotypes of Arabidopsis. We found we could generate Hybrid Mimics by the same repeated selfing/phenotype selection procedure in Ws/L*er* and Col/L*er* crosses. Genes associated with cell wall biosynthesis and expansion were up‐regulated in the hybrids and Mimics of both systems in the rosette leaves of 25‐day‐old plants, indicating up‐regulation of cell wall‐related genes is likely to be a pathway common in the generation of hybrid vigor. Selection for high‐performing Hybrid Mimics and intercrossing of different Mimic lines could be methods for breeding high biomass plants.

## 2 EXPERIMENTAL PROCEDURES

### 2.1 Plant Material and growth conditions

*Arabidopsis* hybrid seeds [Wassilewskija (Ws)/Landsberg *erecta* (L*er*), Col/L*er* and C24/L*er*] were produced by hand‐pollination between parental accessions. Ws/L*er* and Col/L*er* Hybrid Mimic lines and F7 small plant lines were produced from the recurrent selection protocol (Wang et al., 2015, 2017) (Figures S1–S4). Seeds of parental lines, Hybrid Mimics, and small plant lines were obtained through natural pollination without restricting the number of siliques unless specified. In Figure 7, the F1 hybrids (Ws/L*er* and Col/L*er*) and intercross offspring of Hybrid Mimics were produced by hand‐pollination; the silique‐restricting procedure was applied for producing seeds of the control lines: Ws, L*er*, Col, and parental Hybrids Mimics (Meyer, Torjek, Becher, & Altmann, 2004). Sterilized seeds were sown onto plates with Murashige and Skoog (MS) medium (Murashige and Skoog Basal Salts with minimal organics, Sigma‐Aldrich, M6899) supplemented with 3% (wt/vol) sucrose and 0.8% wt/vol agar, pH5.7. Seeds were kept at 4°C for three days in the dark and then transferred into a growth room with conditions of 16‐hr light (22°C)/8‐hr dark (18°C) and light density at 120–150 μmol photons m^−2^ s^−1^. At 15 days after sowing, each plate‐grown seedling was transferred to a 65 mm W × 65 mm L × 100 mm H square pot containing soil (Debco Seed Raising & Superior Germinating Mix, Debco, Australia) and grown in the same growth room [16‐hr light (22°C)/8‐hr dark (18°C); light density: 120–150 μmol photons m^−2^ s^−1^].

### 2.2 Recurrent selection for Hybrid Mimics and small lines

Three hundred Ws/L*er* F2 plants were grown as the selection population. The parents Ws (*n* > 20) and L*er* (*n* > 20) and the reciprocal Hybrids (Ws × L*er* F1: *n* > 20, L*er* × Ws F1: *n* > 20) were grown in the same experiment as controls. For each plant, flowering initiation was scored as the day when inflorescence primordia became visible (IPV). For rosette diameter measurements, plants were photographed with a scale and rosette diameter of each plant was measured using ImageJ software (National Institutes of Health). Selection for an F1‐like phenotype or small plants was performed at 30 DAS based on rosette diameter and the time of flowering initiation. Plants initiating flowering beyond the range of the IPV of parents were excluded from the selection. “F1‐like” is defined as: (a) plants showing IPV within the range of parents' IPV; (b) at 30 DAS plants had the largest rosette diameter in the selection population or at least had rosette diameter similar to the rosette diameter of the F1 hybrids. The smallest plants were selected as the controls of large plants. The same selection process was carried out in Col/L*er* system using 300 Col/L*er* F2 plants and the control lines (Col, L*er*, Col × L*er* F1 and L*er* × Col F1, *n* > 20 per line). Twenty large and seven small F2 plants were selected from Ws/L*er* system (Figures S1 and S3), and 20 large and 10 small F2 plants were selected from Col/L*er* system (Figures S2 and S3). The F3 seeds from each selected F2 plant were harvested separately as different plant lines.

In the selection of the F3 generation, 30 F3 plants from each line were grown for selection, and the recurrent selection process was performed with the largest/smallest plants again selected based on the criteria of flowering initiation time and rosette diameter at 30 DAS. The same selection processes were performed in the F4 and in the subsequent generations. Some F3 plant lines produced by large F2 plants were not selected due to their unsatisfactory phenotype of small plant sizes or flowering initiation time later than both parents in the F3 generation. In the F6, plant lines having a F1‐like phenotypes in rosette diameter and uniformity were termed Hybrid Mimics and used to produce F7 lines. For the growth pattern of the parents, hybrids, and F7 lines, rosette diameters of each plant lines were measured at several time‐points during the growth (10, 15, 20, 25, and 30 DAS). *n* = 12–20.

### 2.3 Germination rate

To minimize the impact of seed age on seed germination, we grew the parental lines (Ws, L*er,* and Col), Ws/L*er,* and Col/L*er* F6 Hybrid Mimics and small plant lines under the same condition. Seeds of each plant were collected at similar time when all siliques were yellow and dry. In the same experiment, crosses between parents (Ws/L*er* and Col/L*er*) were made for hybrid seed production. The rates of seed germination of all plant line (Ws, L*er*, Col, Ws/L*er,* and Col/L*er* F1 hybrids, and Hybrid Mimics and small lines) were examined at approximately 4 weeks after seed collection. At least, 10 seeds per line were scored as one replicate. The data represent the average value from two to four replicates.

### 2.4 Transcriptome sample preparation and RNA extraction

For the sample set of Ws/L*er* system, the rosette leaves of 25‐day‐old parents Ws and L*er*, two reciprocal hybrids Ws × L*er* and L*er* × Ws, seven Hybrid Mimic lines (WL_HM1‐7), and two small lines (wl_sml1‐2) were collected at time Zeitgeber Time (ZT) = 6–8 (ZT = 0 refers to dawn). For the same set of Col/L*er* system, the rosette leaves of 25‐day‐old parents Col and L*er*, two reciprocal hybrids Col × L*er* and L*er* × Col, six Hybrid Mimic lines (CL_HM1‐6), and two small lines (cl_sml1‐2) were collected. The transcriptomes of Hybrid Mimics CL_HM5 and CL_HM6 were excluded from the analysis due to unsatisfactory phenotypes of plant size at sampling day 25 DAS. Three biological replicates were collected per plant line. For the parents and hybrids, rosette leaves from three plants of each genotype were pooled as one biological replicate. For each Hybrid Mimic and small line, rosette leaves from one plant were collected as one biological replicate. RNA was extracted by a Maxwell RSC robot using RNA extraction kit (Promega, Maxwell RSC plant RNA kit, AS1500). The mRNA sequencing was performed by the Novogene [NOVOGENE (HK) COMPANY LIMITE, http://www.novogene.com] with 150 bp paired ends. Raw data of RNA‐seq were deposited in GEO (accession no. GSE131682).

### 2.5 Transcriptome analysis

The quality control reports of each sequencing sample were provided by the sequencing provider. Alignment of sequenced reads was performed using STAR version 2.5.3a against the TAIR10 reference genome and the araport11 annotation. The settings for the sequence alignment are as follows:

- outFilterMismatchNmax 10 \
- outSAMtype BAM SortedByCoordinate \
- quantMode GeneCounts \
- outFilterMultimapNmax 10 \
- outSAMattrIHstart 0 \
- outSAMmapqUnique 255 \
- outSAMmultNmax ‐1 \
- chimSegmentMin 40

More than 90% of reads could be uniquely mapped to exons (Table S2). The mapped counts were normalized according to the library size across all the samples. The DESEQ2 package (Love, Huber, & Anders, 2014) was used to determine significant differences in gene expression between samples under the “RStudio” environment. The detailed scripts are available on request from the corresponding author. A threshold of *p* ≤ .05 was applied to identify differentially expression genes (DEG). A number of DEGs were validated by real‐time PCR. Genes were only considered expressed and analyzed if genes with reads ≥50 across all the samples.

For generating a gene list of shared DEGs in the hybrids and Mimics, two filters were applied: (a) DEGs have to be shared in the F1 hybrids and at least three Hybrid Mimics with the same direction of alteration (up‐regulated or down‐regulated from MPV); (b) genes showing the same up‐ or down‐regulation in the hybrids and both small lines were unlikely to be important for heterotic phenotypes, so were excluded.

### 2.6 Gene ontology (GO) analysis

Gene enrichment analysis was performed on the AgriGO platform version 2.0 (agriGO v2.0: http://systemsbiology.cau.edu.cn/agriGOv2/) (Tian et al., 2017). Genes with reads ≥ 50 at least in one sample were considered expressed genes and used as the background gene list. Significant Go term is defined when significant level <.05 (Statistical test method: Fisher, Multi_test adjustment method: Hocheberg FDR). Minimum number of mapping entries = 5.

### 2.7 Real‐time PCR

RNA samples were treated with DNase I during the RNA extraction process, and then, the products were reverse transcribed using SuperScript® III Reverse Transcriptase (Invitrogen, 18080044). The resulting cDNA was diluted 50–200 times in nuclease‐free water. For real‐time PCR, 10 µl diluted cDNA was used as template in a 20 µl reaction. Real‐time PCR with SYBR green detection was performed using the real‐time PCR instrument ROTOR‐GeneQ (QIAGEN). The expression data for each gene were normalized to the expression of the housekeeping gene *At4g26410* (Czechowski, Stitt, Altmann, Udvardi, & Scheible, 2005). The following primers were used:

*CesA1‐F: 5′‐ GCTGTAACAAGGGGAGGCTC ‐3′*
*CesA1‐R: 5′‐ CCTATCCCATCTTCTCGCCTAACC ‐3′*
*UGD4‐F: 5′‐ CGTTTTGACTGAGTGGGACGAGT‐3′*
*UGD4‐R: 5′‐ GACAAAGGCAGGCATGTCCTTG ‐3′*
*XTH4‐F: 5′‐ TGGCGGTTCCGAACTTCAGC ‐3′*
*XTH4‐R: 5′‐ GGTTGTCCTGTTCTGTTTCCAAGGA ‐3′*
*XTH8‐F: 5′‐ CATCGGCTTCTGGTTCGGGA ‐3′*
*XTH8‐R: 5′‐ GGAATCGGGAGATGCGACATTCC ‐3′*
*AT4G26410‐F: 5′‐ GAGCTGAAGTGGCTTCCATGAC ‐3′*
*AT4G26410‐R: 5′‐ GGTCCGACATACCCATGATCC ‐3′*

## 3 RESULTS

### 3.1 Hybrid Mimics were selected from Ws/L*er* and Col/L*er* hybrids

**Figure 1:**
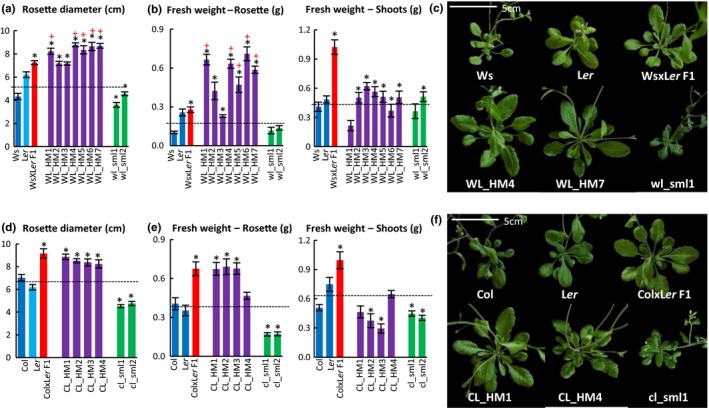
Hybrid Mimics selected from Ws/L*er* and Col/L*er* systems had increased rosette sizes and fresh weight at 30 days after sowing (DAS). Rosette diameter (RD) (a) and fresh weight (FW) (b) of the parents Ws and L*er*, WsxL*er* hybrids, seven F7 Ws/L*er* Hybrid Mimic lines (WL_HM 1‐7), and two F7 small lines (wl_sml1‐2) selected from Ws/L*er* system at 30 DAS. (c) Rosette phenotypes of the parents Ws and L*er*, Ws/L*er* hybrids, two representative Hybrid Mimic lines (WL_HM 4 and 7), and one small line (wl_sml1) at 30 DAS. Scale bar = 5 cm. Rosette diameter (d) and fresh weight (e) of parents Col and L*er*, ColxL*er* hybrids, four Col/L*er* Hybrid Mimic lines (CL_HM 1–4), and two small lines (cl_sml1‐2) selected from Col/L*er* system at 30 DAS. (f) Rosette phenotypes of the parents Col and L*er*, Col/L*er* hybrids, two representative Hybrid Mimic lines (CL_HM 1 and 4), and one small line (cl_sml1) at 30 DAS. Scale bar = 5 cm. The black dotted line represents MPV. For the fresh weight measurements, the fresh weights of rosette leaves and shoots of each plant were measured separately. *p* value is generated using Student's *t* test. * indicates *p* < .05; + indicates RD/FW > F1, *p* < .05. Error bars = *SE*, *n* > 7

**Figure 2:**
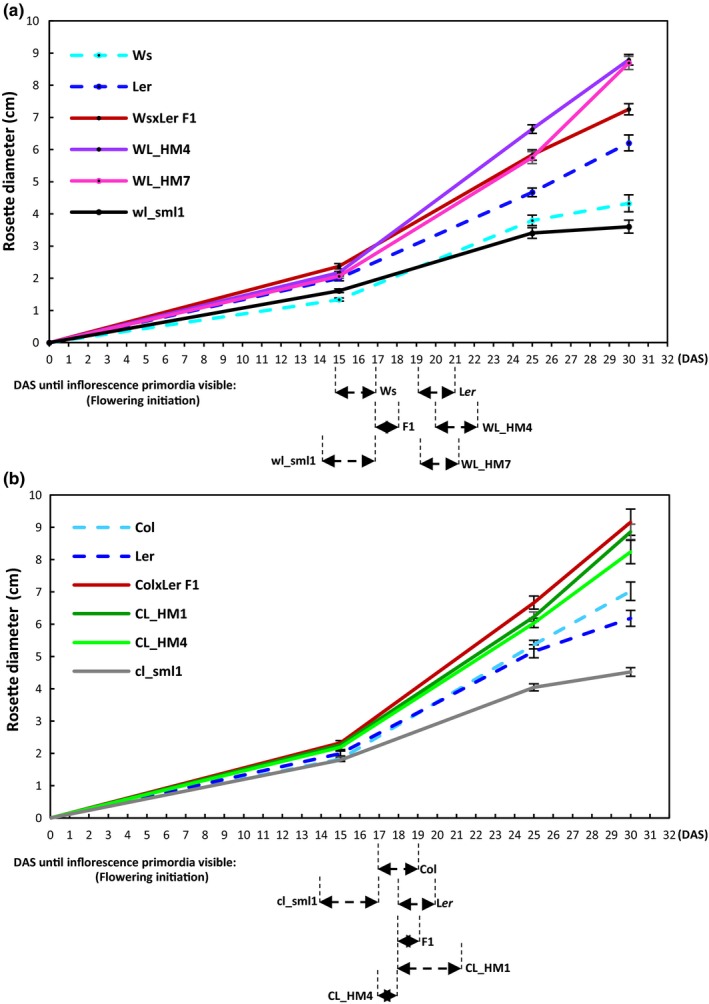
Hybrid Mimics selected from Ws/L*er* and Col/L*er* systems showed growth patterns similar to the hybrids. (a) Growth course of parent Ws and L*er*, WsxL*er* hybrids, two representative Hybrid Mimic lines (WsL*er*_HM 4 and 7), and one small line (wsl*er*_sml1). Error bars = *SE*, *n* = 12–15. (b) Growth course of parent Col and L*er*, ColxL*er* hybrids, two representative Hybrid Mimic lines (ColL*er*_HM 1 and 4), and one small line (coll*er*_sml1). Error bars = *SE*, *n* = 17–20. The time of flowering initiation was scored as the DAS until inflorescence primordia were visible. The time frame of flowering initiation in each line was marked under the graph

Repeated F1‐like phenotype selection in the Ws/L*er* and Col/L*er* systems resulted in F6/F7 Hybrid Mimics in both hybrid systems (Figure 1a‐f and Figures S1–S4). Hybrids and Hybrid Mimic lines showed similarity in growth patterns as measured by increased rosette diameter compared with MPV (Figure 2a,b and Figure S5a,b). As in the C24/L*er* hybrids (Zhu et al., 2016), both Ws/L*er* and Col/L*er* hybrids germinated earlier than the parent lines (Table 1). At 28 hr after sowing (HAS), 90% of Col/L*er* hybrid seeds had germinated, approximately 3 hr ahead of the seeds of parental lines. At 24 HAS, 100% Ws/L*er* hybrids seeds had germinated, approximately 7 hr ahead of the seeds of parental lines. Earlier germination occurred in three of the Col/L*er* Hybrid Mimics (3 of 4) and five of the Ws/L*er* Hybrid Mimics (5 of 7); the small plant lines used as a control were similar to the parents in germination time (Table 1).

**Table 1:** Germination time of the parents (Ws, L*er,* and Col), hybrids (Ws/L*er* and Col/L*er*), Hybrid Mimics, and small lines

	Ws (%)	L*er* (%)	Ws/L*er* F1 (%)	WL_HM1 (%)	WL_HM2 (%)	WL_HM3 (%)	WL_HM4 (%)	WL_HM5 (%)	WL_HM6 (%)	WL_HM7 (%)	wl_sml1 (%)	wl_sml2 (%)
24 HAS	45	29	100	88	81	93	64	80	87	58	29	36
28 HAS	66	57	100	88	94	100	86	88	100	94	71	50
31 HAS	100	85	100	94	100	100	100	88	100	100	93	100
48 HAS	100	100	100	100	100	100	100	100	100	100	100	100

	Col (%)	L*er* (%)	Col/L*er* F1 (%)	CL_HM1 (%)	CL_HM2 (%)	CL_HM3 (%)	CL_HM4 (%)		cl_sml1 (%)	cl_sml2 (%)		
24 HAS	60	29	68	87	60	31	94		39	14		
28 HAS	77	57	90	93	67	86	100		79	35		
31 HAS	100	85	95	93	87	100	100		100	47		
48 HAS	100	100	100	100	100	100	100		100	100		

Note

The values present the percentage of germinated seeds at 24, 28, 31, and 48 hr after sowing (HAS). The red type indicates the time point when the majority of seed from a line (>80%) have germinated.

Ws/L*er* and Col/L*er* F1 hybrids had rosette diameters 20% and 15% larger than the better parent (L*er*) at 15 DAS (Figure S5c). Four Hybrid Mimics (WL_HM2, 3, 4, and 5) had rosette diameter similar to the Ws/L*er* hybrids, and WL_HM1, 6, and 7 had rosette sizes similar to the parent L*er* at 15 DAS (Figure S5c) (*p* > .05). The hybrids and Mimics had rapid growth rates at approximately two to three weeks after sowing and at later stages (Figure 2a,b and Figure S5a,b). At 30 DAS, all seven Ws/L*er* Hybrid Mimic lines had larger rosette diameters than the MPV (Figure 1a,c) (*p* < .05). In the Col/L*er* system, the hybrids had increased total fresh weights; the four Hybrid Mimics had fresh weights similar to the larger parent (L*er*) (Figure 1e) (*p* > .05)*.* At 30 DAS, plants were at different developmental stages due to flowering‐time differences. The ratio between the biomass of rosette leaves and shoots was different in the parents, hybrids, and F7 lines (Figure 1b,e).

Five Mimic lines in the Ws/L*er* system (WL_HM1, 4, 5, 6, and 7) had rosette diameters larger than the F1 with 13%–21% increase and 169%–255% increase in the fresh weight of the rosette leaves (*p* < .05). The remaining two Hybrid Mimic lines WL_HM 2 and 3 were equivalent to the F1 in plant size (Figure 1a,b). The overall fresh weights of WL_HM lines were less than the Ws/L*er* hybrid due to a less well‐developed shoot at 30 DAS, since the WL_HMs had delayed flowering initiation relative to the Ws/L*er* F1 hybrids (Figure 1b). Hybrid Mimic lines had a high level of uniformity in growth pattern, rosette size, leaf morphology, and flowering time, indicating a “fixed” phenotype; the uniformity of WL_HM5 and WL_HM7 was lower than the F1 hybrids and other Hybrid Mimics (Figures S4–S6), Table S1).

In the Col/L*er* system, the two earlier germinating Hybrid Mimics had plant sizes comparable to the F1 and larger than the parents at 15 DAS (Figure S5c). The remaining two HMs (CL_HM2 and 3) had rosette diameters similar to the parents at 15 DAS (Figure S5c) (*p* > .05). Unlike the Hybrid Mimic selections in Ws/L*er*, none of the four CL_HMs had rosette diameters or fresh weights greater than the hybrid (Figure 1d,e). The two small F7 lines in the Col/L*er* system (cl_sml1‐2) had plant size and rosette biomass approximately half to two‐thirds that of the parents (Figure 1a–f).

### 3.2 Identification of differentially expressed genes in the Hybrid and Mimics

**Figure 3:**
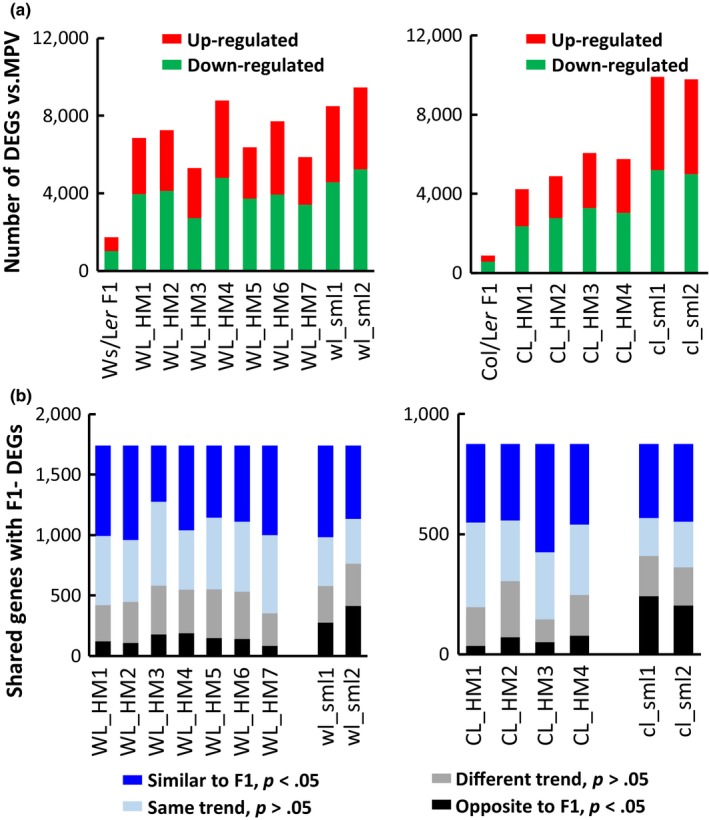
Transcriptome analysis of parents, hybrid, and hybrid mimics. (a) Numbers of differentially expressed genes (DEGs) in the hybrids, Mimics, and small lines compared with the MPV. Red and green colors indicate the up‐/down‐regulated genes. (b) The number of shared DEGs between F1 and Hybrid Mimic or small line. F1 DEGs were categorized into four groups based on the expression patterns and the *p* value from the MPV in the F7 line

Rosette leaves of 25 DAS plants from each Hybrid Mimic, parents, F1 hybrids, and small lines were sampled for transcriptome analysis. Principal component analysis (PCA) of the transcriptome data showed similarity of the gene expression patterns in hybrids and Mimics (Figure S7). Transcriptomes of each of the Hybrid Mimic lines were compared with the corresponding parents and hybrids (Table S2). Approximately 18,000 genes were expressed with a read cut‐off of 30. Differentially expressed genes (DEG) were defined by a significance value of *p* < .05 from the MPV. A total of 8,681 genes, approximately 50% of expressed genes, were differentially expressed between Ws and L*er*; fewer DEGs (2,053 genes, 11%) were identified between Col and L*er*. 1,740 DEGs (9.5%) were identified in the Ws/L*er* hybrids compared with the MPV (Figure 3a, Table S3). Of the 1,740 DEGs, approximately half (782 genes) were differentially expressed between the two parents (Table S3). The numbers of differentially expressed genes in the Ws/L*er* Hybrid Mimics and small lines ranged from 5,310 to 9,459 (29% −52%) (Figure 3a). Of the 876 DEGs (5% of the expressed genes) in the Col/L*er* hybrids, 171 genes were differentially expressed between the two parents (Figure 3a, Table S3). The numbers of DEGs in different Col/L*er* F7 lines ranged from 4,226 to 9,906 (23% −55%) (Figure 3a). In both Ws/L*er* and Col/L*er* hybrids, approximately two‐thirds of DEGs were down‐regulated compared with the MPV (Figure 3a, Table S3). In each F7 Hybrid Mimic line, there were more DEGs down‐regulated than up‐regulated (Figure 3a).

In the Hybrid Mimics, the majority (65%–83%) of F1 DEGs had differential expression in the same direction or showed the same trend; only a small proportion (4%–11%) of F1 DEGs was expressed in an opposite direction. In the small lines, 16%‐28% of F1 DEGs were expressed in an opposite direction (Figure 3b).

The DEGs common to F1 hybrids and Hybrid Mimic lines are likely to be associated with their common phenotypes of increased rosette size and biomass. Of the 550 DEGs shared between the Ws/L*er* hybrids and three or more Mimic lines but not shared with the two control small lines, genes in the GO terms “cell wall organization or biogenesis” (37 genes) and “defense response” (49 genes) were overrepresented (Figure S8, Tables S4 and S5). In the Col/L*er* set (179 DEGs), there was significant enrichment of the DEGs in pathways including “cell redox homeostasis” (7 genes encoding glutaredoxin) and “flowering time, shoot system development” (13 genes) (Figure S8, Tables S6 and S7).

### 3.3 Up‐regulation of cell wall biosynthesis in the hybrids and Hybrid Mimics

**Figure 4:**
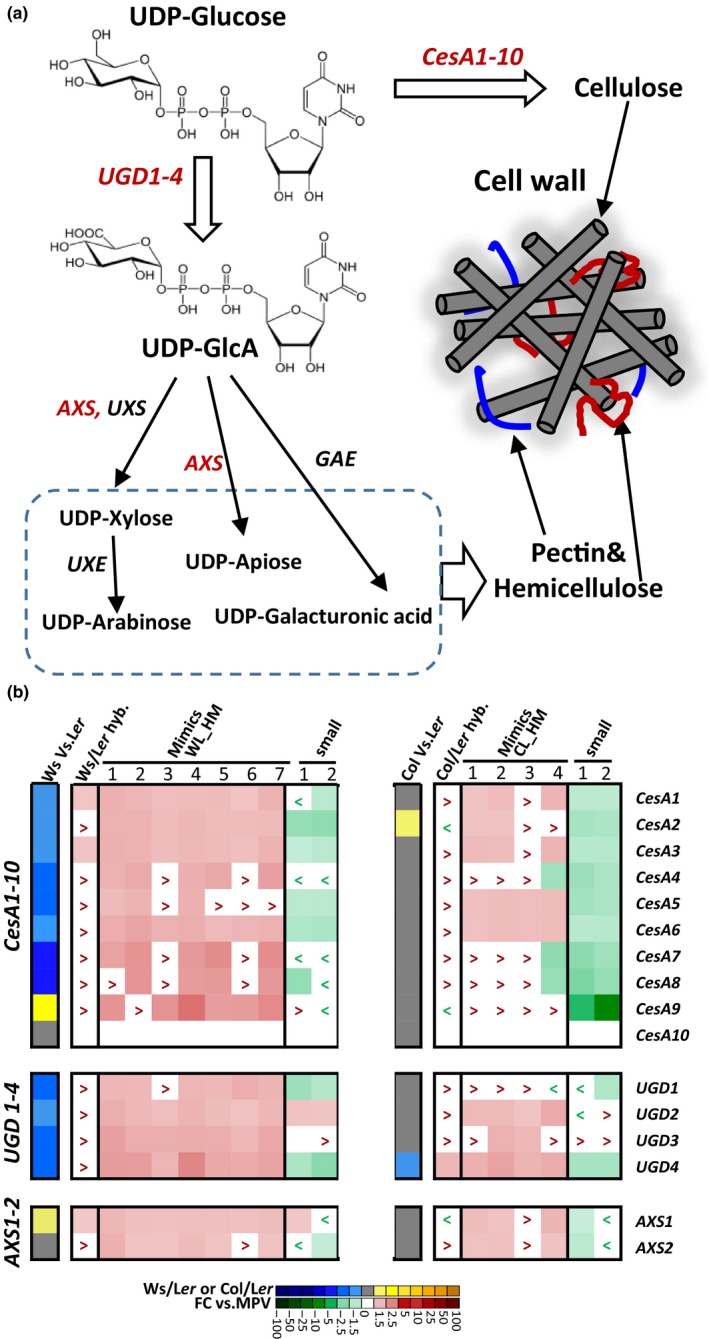
Cell wall biosynthesis genes were up‐regulated in the hybrids and Mimics. (a) The biochemical pathway of cell wall biosynthesis. Genes encoding the enzymes for production of cellulose, hemicellulose, and pectin are indicated. Genes up‐regulated in the hybrids and Mimics are in red letters. UDP‐GlcA, UDP‐glucuronic acid; *CesA: cellulose synthase*; *UGD: UDP‐glucose dehydrogenase*; *AXS, UDP‐apiose/UDP‐xylose synthase; UXS, UDP‐xylose synthase; GAE, UDP‐glucuronic acid epimerase; UXE, UDP‐xylose epimerase*. (b) Heat maps showing the expression levels of cell wall biosynthesis genes *CesA1‐10*, *UGD1‐4*, and *AXS1‐2* in the F1 hybrids, Hybrid Mimics, and small lines in Ws/L*er* and Col/L*er* systems. Red/green colors indicate the up‐/down‐regulated fold change (FC) from the MPV. “>” or “<” indicates that expression shows an up or down trend, but the change is not significant (*p* > .05 from the MPV). Blue/yellow color indicates the comparison between the two parents in Ws/L*er* or Col/L*er* system

Of the 37 DEGs annotated in “cell wall organization or biogenesis”, 32 were up‐regulated in the Ws/L*er* hybrids and Hybrid Mimics, indicating increased activity of cell wall biosynthesis and/or cell wall expansion genes (Table S8). Cellulose, the primary component of cell walls, is synthesized by cellulose synthase (CesA) complexes (Endler & Persson, 2011). Arabidopsis contains 10 cellulose synthases (CesA1‐10) (McFarlane, Doring, & Persson, 2014). We found that apart from *CesA10* which was expressed at a low level in leaf tissue, all nine other *CesA* genes (*CesA1‐9*) were significantly up‐regulated or showed a trend of up‐regulation in the Ws/L*er* hybrids and the seven Hybrid Mimics (Figure 4a,b). Both *CesA1* and *CesA3* had 15%‐55% higher levels of expression in the Ws/L*er* hybrids, and the seven Hybrid Mimics were compared with the MPV. Five *CesA* genes had reduced expression in the small plants (Figure 4b). In the small line wl_sml1, *CesA3* was down‐regulated by 20%. In the second small line (wl_sml2), both *CesA1* and 3 were more than 30% down‐regulated compared with the MPV (Figure 4b).

In the Ws/L*er* hybrids and Mimics, changes in gene expression were also found in the biosynthesis of non‐cellulosic wall polysaccharides. UDP‐glucose dehydrogenase (UGD) plays a key role in the nucleotide sugar biosynthetic pathway. UGDs convert UDP‐glucose to UDP‐glucuronic acid (UDP‐GlcA), which is a common precursor of arabinose, xylose, galacturonicacid, and apiose residues, the substrates for hemicellulose and pectin (Klinghammer & Tenhaken, 2007) (Figure 4a). Approximately 50% of cell wall biomass is derived from the precursor UDP‐GlcA (Zablackis, Huang, Muller, Darvill, & Albersheim, 1995), and the activities of all four *UGD* genes were significantly increased in Ws/L*er* Hybrid Mimics, although the up‐regulation of *UGD*s was not as marked in the Ws/L*er* F1 hybrid. *UGD1* and *4* were down‐regulated more than 50% in the two small plant lines (wl_sml1 and 2) (Figure 4b).

*UDP‐D‐APIOSE/UDP‐D‐XYLOSE SYNTHASE 1 (AXS1)* is involved in cell wall biosynthesis by catalyzing the conversion of UDP‐D‐glucuronate to a mixture of UDP‐D‐apiose and UDP‐D‐xylose (Ahn et al., 2006). *AXS1* was up‐regulated in the Ws/L*er* hybrids and all seven Mimics, but not in the small line wl_sml2. *AXS2* showed gene activity similar to *AXS1* with an increased number of transcripts in the hybrid and Mimics, and fewer in the small plants (Figure 4b). Cell wall biosynthesis genes were more up‐regulated or trended to up‐regulation in the Ws/L*er* Hybrid Mimics than in the F1 hybrid, correlating with the plant sizes of Mimics being larger than the F1 hybrids (Figures 1 and 4).

The Col/L*er* hybrids and Mimics had the expression of the cell wall biosynthesis genes, *CesA, UDG,* and *AXS*, showing a trend of up‐regulation similar to the changes in the Ws/L*er* Hybrid and Mimics (Figure 4b). Nine *CesAs* were down‐regulated in the small plants selected from the Col/L*er* combination (Figure 4b). The key cell wall biosynthesis gene *UDG4* had 42% increased expression in the F1 and 50%‐80% up‐regulation in Hybrid Mimics. In both small lines cl_sml1 and 2, the majority of cell wall biosynthesis genes were down‐regulated (Figure 4b).

In crosses between the C24 and L*er* ecotypes, the F1 hybrids had substantial levels of hybrid vigor in vegetative biomass and plant size (Groszmann et al., 2014). In the Hybrid Mimic line L2 (referred as HM‐G here) selected from the C24/L*er* hybrid system (Wang et al., 2015), at 28 days the cell wall biosynthesis genes *CesA*, *UDG,* and *AXS* had higher levels of transcripts in the rosette leaves of F1 hybrids and Mimics than the MPV (Wang et al., 2015, Figure S9).

### 3.4 Up‐regulation of *XYLOGLUCAN ENDOTRANSGLUCOSYLASE (XTH)* genes in the Hybrid and Mimics

**Figure 5:**
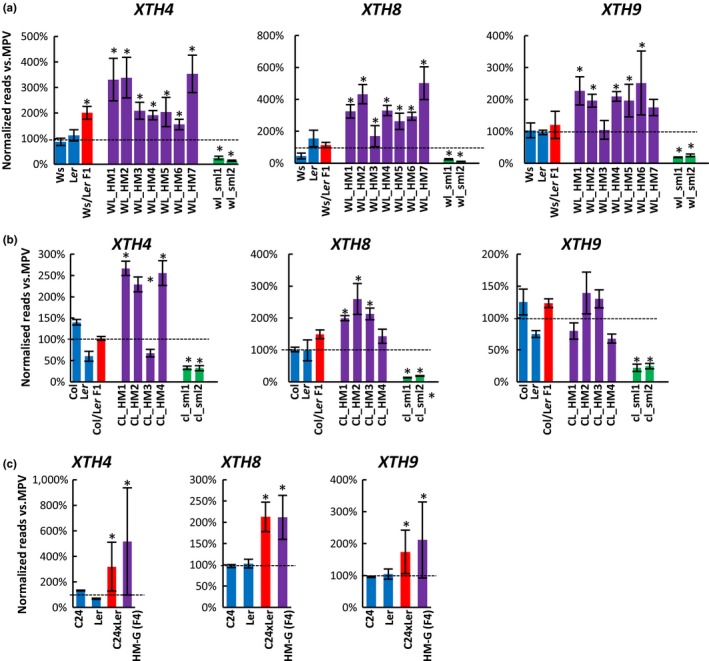
Examples of up‐regulated *XTH* genes in the hybrids and hybrid mimics. (a–b) Relative expression of *XTH4, XTH8,* and *XTH9* in the parents (Ws, L*er,* and Col), hybrids (Ws/L*er* and Col/L*er*), Hybrid Mimics (WL_HM1‐7, CL_HM1‐4), and small lines (wl_sml11‐2 and cl_sml1‐2)*.* For each gene, the expression levels in each plant line were normalized to MPV by setting the MPV as “1.” The data represent the mean of biological replicates (*n* ≥ 3). (c) Relative expression of *XTH4, XTH8,* and *XTH9* in the 28 DAS rosette leaves of parents C24 and L*er*, C24xL*er* hybrids, and F4 Hybrid Mimics (HM‐G) (Wang et al., 2015)*.* The mean of normalized reads from biological replicates (*n* = 2) of each line was normalized to the MPV by setting the MPV as “1.” The black dotted line represents MPV. * indicates significant differences at *p* (Student's *t* test) <.05 from MPV. Error bars = *SE*

XTHs cut and ligate xyloglucans as a means of integrating new xyloglucans into the cell wall and are important for loosening existing wall material and enabling cell expansion (Becnel, Natarajan, Kipp, & Braam, 2006; Rose, Braam, Fry, & Nishitani, 2002). The Arabidopsis genome has 33 *XTH* genes, 25 of which are expressed in 25 DAS rosette leaves. Six *XTH* genes were up‐regulated in the Ws/L*er* F1 hybrids. Six to nine *XTH* genes were up‐regulated in the seven Ws/L*er* Hybrid Mimic lines with two to four *XTH* genes overlapping the up‐regulated *XTH*s in the F1 hybrid. Of the eight up‐regulated *XTH* genes in WL_HM7, four *XTHs* were also up‐regulated in the hybrid. In the two small control lines, half of the expressed *XTH* genes had decreased transcript levels or showed a trend of down‐regulation relative to MPV (Figure 5a,b, Table S9). The up‐regulation of four cell wall‐related genes in Ws/L*er* Hybrid and Mimics was validated by quantitative real‐time PCR (Figure S10).

Three *XTH* genes (*XTH4, XTH8,* and *XTH9*) were up‐regulated or had a trend of up‐regulation in two other Hybrids (Col/L*er* and C24/L*er*) and in the Mimic lines; these loci were not up‐regulated in the small plants (Figure 5c and Figure S9).

### 3.5 The defense response pathway genes were down‐regulated in both hybrids but not in all Mimics

Down‐regulation of defense response genes has been reported to result in the up‐regulation of growth genes and contribute to heterosis in hybrids having C24 as one parent (Gonzalez‐Bayon et al., 2019; Groszmann et al., 2015; Miller, Song, Shi, Juenger, & Chen, 2015; Wang et al., 2015). In the rosette leaves of 25‐day‐old Ws/L*er* hybrids, 49 DEGs were associated with “defense response”; the majority (35 genes) were down‐regulated in the F1 hybrids and Mimics (Table S10). The 35 down‐regulated defense response‐associated genes included genes encoding disease resistance receptor (PRR/PRR/NLRs) proteins (Glowacki, Macioszek, & Kononowicz, 2011), protein kinases (Wang, Schuck, et al., 2018; Xu et al., 2016), disease‐related transcription factors WRKYs (Eulgem, Rushton, Robatzek, & Somssich, 2000), and downstream defense‐responsive genes (Table S10).

**Figure 6:**
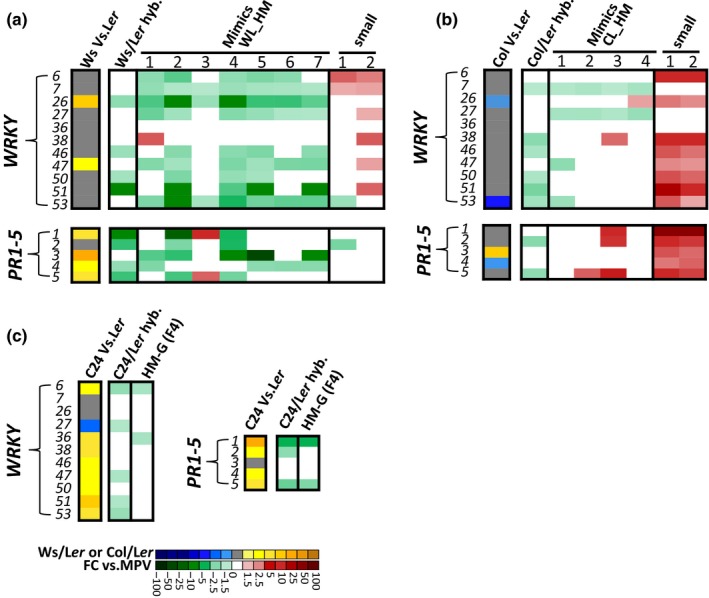
The defense response pathway was down‐regulated in both hybrids but not in all Mimics. Heat maps showing the expression levels of WRKY genes (a) and PR genes (PR1‐5) (b) in the Ws/Ler and Col/Ler hybrid, Hybrid Mimics (WL_HM1‐7 and CL_HM1‐4), and small lines. (c) The down‐regulation of defense response genes WRKYs and PR genes in the 28 DAS rosette leaves of C24/L*er* hybrids and F4 Hybrid Mimic line G (HM‐G). Different red/green colors indicate the up‐/down‐regulated fold change from the MPV. Blue/yellow color indicates the comparison between the two parents in Ws/L*er* or Col/L*er* system

Four *WRKY* genes (*WRKY26, 46, 50, and 51*) were down‐regulated in the Ws/L*er* hybrids, and three or more *WRKY* genes were down‐regulated in the Mimics (Figure 6a, Table S11). *WRKY* genes have roles in regulating pathogen‐induced defense responses (Eulgem et al., 2000). Of the 72 *WRKY*s in the Arabidopsis genome, the overall level of gene activities of *WRKY*s was down‐regulated in the Ws/L*er* F1 and in the Mimics, but they were up‐regulated in the small plant lines (Table S11), consistent with the concept of a trade‐off between plant growth and the level of defense response gene expression (Denance, Sanchez‐Vallet, Goffner, & Molina, 2013).

The Arabidopsis genome contains five *PATHOGENESIS‐RELATED (PR)* genes (Mishina & Zeier, 2006). In the Ws/L*er* system, four *PR* genes (*PR1, 2, 4, and 5*) were down‐regulated in the F1 hybrids. Six of the seven Hybrid Mimics had decreased *PR* expression (Figure 6a). In the Hybrid Mimic with the lowest biomass (Figure 1b), WL_HM3, two *PR* genes *PR1,* and *PR5* were up‐regulated (Figure 6a). The small plant line wl_sml1 had down‐regulation of the *PR2* gene, but *PR* genes were not down‐regulated in wl_sml2 (Figure 6a).

In the Col/L*er* hybrid system, the majority of the defense response genes were down‐regulated in the F1 hybrids. In the Mimics, most defense response genes were expressed at MPV. Both small lines had defense genes up‐regulated with all five *PR* genes expressed at least twofold higher than the MPV (Figure 6b). *WRKY* genes and *PR* genes were down‐regulated in the 28‐day rosette leaves of hybrid and Mimics (G line) in the C24/L*er* system (Figure 6c).

Some genes in the defense response pathway are also involved in the leaf senescence pathway (Gonzalez‐Bayon et al., 2019). Senescence genes had decreased expression in the Ws/L*er* Hybrid Mimics and increased transcript levels in one Ws/L*er* small line. The up‐regulation of senescence genes occurred in both Col/L*er* small lines (Figure 6 and Figure S11).

### 3.6 Flowering genes play a role in producing the large rosette phenotype

Flowering plays an important role in plant growth and development in Arabidopsis (Jung, Pillen, Staiger, Coupland, & Korff, 2017). Plants with later flowering times are more likely to have larger plant sizes due to the longer vegetative phase. In the recurrent selection processes, the initiation of flowering of each plant was scored as the day when flower buds were first visible in the center of the rosette. The selection for both large and small phenotypes was subject to the criterion of flowering times being within the range of the two parents. During the selection process from the F2 to the F5 generation, the flowering times of large and small plants diverged: plants selected for the small phenotype had a flowering time similar to the early‐flowering parent or even 1–2 days earlier, while plants with larger rosette sizes had flowering times close to the later flowering parent (Table S1).

Genes in “regulation of shoot system development” and “regulation of flower development” were enriched in the DEGs shared by the Col/L*er* F1 hybrids and four Mimics (Figure S8). In Arabidopsis, the flowering‐time pathway contains at least three genes that are major regulators of flowering: *SUPPRESSOR OF OVEREXPRESSION OF CONSTANS 1 (SOC1*, or *AGL20*), *FLOWERING LOCUS T (FT),* and *LEAFY (LFY)* (Boss, Bastow, Mylne, & Dean, 2004; Simpson & Dean, 2002). In our datasets, *LFY* was expressed at a low level making a change in its gene expression difficult to score. In agreement with the Hybrid Mimics having a slight delay of flowering initiation (Table S1), *FT* and *SOC1* were down‐regulated in the two sets of Hybrid Mimics (Figure S12). Down‐regulation of the flowering associated genes also occurred in the Ws/L*er* and Col/L*er* F1 hybrids (Figure S12).

### 3.7 Intercrosses of Hybrid Mimics

In the Ws/L*er* Hybrid Mimics, cell wall‐related genes were up‐regulated, but the number of up‐regulated cell wall‐related genes and their levels of up‐regulation were not the same in the different lines (Figure 4b). In the Ws/L*er* Hybrid Mimic, plant defense pathway genes were down‐regulated, but the same genes were not significantly altered in the Col/L*er* Hybrid Mimics (Figure 4b). Hybrid Mimics may differ in growth pattern and rosette size as a result of different genes or pathways operating in each different Hybrid Mimic line.

**Figure 7:**
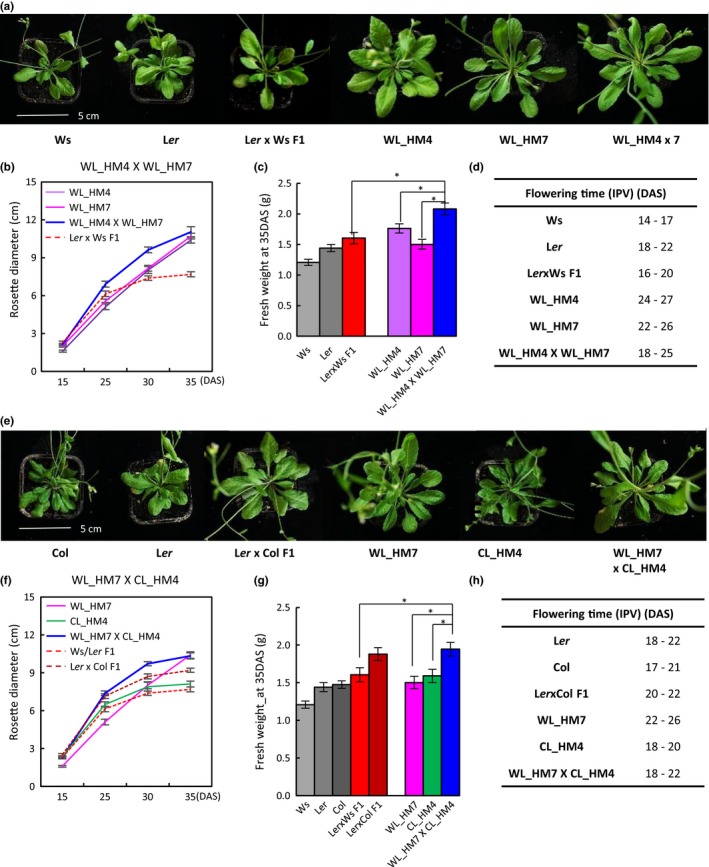
Intercrossing of Hybrid Mimics increases growth vigor. (a) Photographs showing the rosette diameters of two parents Ws and L*er*, Ws/L*er* F1 hybrids, two Hybrid Mimics WsL*er*_HM4, and 7 and intercross progeny (WsL*er*_HM7 × 4) at 35 DAS. (b) Intercross progeny showed increased rosette diameter during the plant growth. Rosette diameters were measured at 15, 25, 30, and 35 DAS. (c) Fresh weights of two parents Ws and L*er*, F1 hybrids, two Hybrid Mimics WsL*er*_HM4 and 7, and intercross progeny (WsL*er*_HM7 × 4) at 35 DAS. (d) Time of flowering initiation (DAS until inflorescence primordia visible, IPV) of parents Ws and L*er*, F1 hybrids, two Hybrid Mimics WsL*er*_HM4 and 7, and intercross progeny (WsL*er*_HM7 × 4). (e) Photographs showing the rosette diameters of two wild‐type parents Col and L*er*, Col/L*er* hybrids, two Hybrid Mimics WsL*er*_HM7 and ColL*er*_HM4, and intercross progeny (WsL*er*_HM7 × ColL*er*_HM4) at 35 DAS. (f) Intercross progeny showed increased rosette diameter during the plant growth. Rosette diameters were measured at 15, 25, 30, and 35 DAS. (g‐h) Fresh weights at 35 DAS and time of flowering initiation (IPV) of two wild‐type parents Col and L*er*, Col/L*er* hybrids, two Hybrid Mimics WsL*er*_HM7 and ColL*er*_HM4, and intercross progeny (WsL*er*_HM7 × ColL*er*_HM4). Error bars = *SE*. *n* > 10. *indicates significant differences at *p* (Student's *t* test) <.05. Scale bar (5 cm) apply to (a) and (e)

The progeny of the intercrosses between WL_HM4 and WL_HM7 had rosette sizes larger than the parental Hybrid Mimics (Figure 7a–c). Flowering of the intercross plants WL_HM4 × 7 was slightly earlier than the parental Hybrid Mimics (Figure 7d). At 35 DAS, the intercross offspring had rosette diameters similar to the better parental Hybrid Mimic line WL_HM7 (*p* > .05). The fresh weight of intercross offspring was increased by 20% compared with the better parental Hybrid Mimic and was 30% greater than the L*er* × Ws F1 hybrid, and comparable to L*er* × Col F1 (Figure 7a–c).

In crosses between Ws/L*er* Hybrid Mimic WL_HM7 and CL_HM4, the offspring initiated flowering at 18–20 DAS, earlier than the WL_HM7 line [DAS until inflorescence primordia were visible (IPV): 22–26 DAS] (Figure 7e–h). The offspring had plant sizes similar to the better parental Hybrid Mimic CL_HM4 at 15 DAS (*p* > .05), followed by a rapid growth period with increased rosette size at 25 and 30 DAS compared with the better parental Hybrid Mimics and Ws/L*er* hybrids (Figure 7f). At 35 DAS, the fresh weights of the intercross offspring were 20% larger than the better parental Hybrid Mimic CL_HM4 and the Ws/L*er* hybrids (Figure 7g). Data from the other two crosses (CL_HM1x4 and WL_HM4 × CL_HM1) showed similar results in increased fresh weight at 35 DAS compared with parental Hybrid Mimic lines (Figure S13).

In crosses made between Hybrid Mimics within the Ws/L*er* hybrid system, plant size could be greater than the F1, presumably resulting from a new combination of genomic segments contributing to the vegetative growth with some new pathways and gene interactions. In mature, postflowering plants, some of the Hybrid Mimics, particularly in the Ws/L*er* system, had rosette diameters larger than the F1 hybrid. Crosses between Hybrid Mimics from the two hybrid systems generated plants having greater rosette diameters and biomass than either of the parental Hybrid Mimics.

## 4 DISCUSSION

The Hybrid Mimics and hybrids of both hybrid systems all germinated earlier than the parents or small lines (Table 1). In both the Ws/L*er* and Col/L*er* systems, there was no correlation between rosette diameter at 30 DAS and timing of germination (Figure 1a,d, Table 1). At 15 DAS, the earlier germinating Col/L*er* Hybrid Mimics were larger in rosette diameter than other Col/L*er* Hybrid Mimics suggesting the earlier germination results in early vegetative hybrid vigor (Figure S5c, Table 1). In the Ws/L*er* system, the hybrids and Mimics germinated at a similar time, and so at 15 DAS, there was little difference between lines. The parents and small lines germinated later and showed less vigor at 15 DAS. The early germination seen in Mimics and hybrids occurs in other hybrids, for example in maize; vigorous hybrids with heterosis at maturity in term of height and yield of grain germinated earlier than non‐vigorous hybrids (Sarkissian, Harris, & Kessinger, 1964).

The early germination of hybrid seeds is likely to be due to heterosis occurring during embryogenesis (Alonso‐Peral et al., 2017). The two genomes in the hybrid may interact as early as the single‐cell zygote stage to produce more vigorous growth and development during embryogenesis priming the seeds for more rapid germination. In our experiments, the rates of seed germination were examined on Murashige and Skoog (MS) medium supplemented with 3% (wt/vol) sucrose using freshly collected seeds (approximately 4 weeks after seed collection). On moist soil, C24/Col hybrids had germination times similar to the faster germinating parent Col (48 hr after sowing), while parent C24 germinated approximately 20 hr later (Meyer et al., 2012). The observations of different germination times of Arabidopsis hybrids compared with their parents can be due to differences in growth condition, the age of the seeds, or different hybrid genotypes.

### 4.1 Common pathways up‐regulated in all three hybrid systems

Plant cell walls are composed primarily of cellulose associated with hemicelluloses and pectin (Thompson, 2005). Cell wall genes were up‐regulated at later stages of growth in the Ws/L*er* and Col/L*er* hybrid systems as well as in the previously studied C24/L*er* hybrid but not in the small plant lines also selected from F2 plants. The Arabidopsis genome contains 10 *CELLULOSE SYNTHASE (CesA)* genes. Mutation of a single *CesA* gene does not necessarily result in a new phenotype but some double or triple mutants in different *CesA* genes have a dwarf or lethal phenotype, emphasizing the critical role of *CesA* loci in plant growth and biomass (McFarlane et al., 2014). Transgenic plants overexpressing *CesA*2, 5, or 6 were taller than the wild type and produced 20% more biomass in 7‐week‐old mature plants (Hu et al., 2018). *UDG* genes were up‐regulated in each of the hybrids and Mimics. These genes are important in specifying plant size. The double mutant *ugd2 and 3* lacking two of the four *UGD* genes has a dwarf phenotype (Reboul et al., 2011). Transgenic *Arabidopsis* plants overexpressing a *UGD* ortholog from *Larix gmelinii* has an increased content of hemicelluloses and enhanced vegetative growth (Li, Chen, et al., 2017).

Another contributor to plant cell wall biology is the *XTH* gene family. A number of *XTH* genes are up‐regulated in all three hybrid systems but not in the small plant lines. *XTH* gene products participate in cell wall growth and remodeling by endolytically cleaving xyloglucan polymers and joining the newly generated end to another xyloglucan chain in the plant cell wall (Rose et al., 2002). The Arabidopsis genome encodes 33 *XTH* genes expressed in every developmental stage from seed germination through flowering (Becnel et al., 2006).

### 4.2 Hybrid Mimics differ in defense response pathway genes

In C24/L*er* F1 hybrids, changes in defense and stress response gene expression are consistent with a reduction in transcription of basal defense genes (Groszmann et al., 2015; Miller et al., 2015). The decreased expression of defense response genes may contribute to the increased growth of the hybrids compared with the average growth of parents through changes in expression of the regulator *ARABIDOPSIS THALIANA CLASS B HEAT SHOCK FACTOR B1 (TBF1)* (Gonzalez‐Bayon et al., 2019; Pajerowska‐Mukhtar et al., 2012), which is involved in the control of the balance between growth and defense. C24 has high levels of salicylic acid (SA) which do not affect its growth but hybrids with C24 as one parent have a decreased level of SA relative to C24 and down‐regulated defense pathway genes (Bechtold et al., 2010; Groszmann et al., 2015). In Ws/L*er* and Col/L*er* hybrids, there is little difference between hybrids and parents in SA level; both hybrids have some down‐regulated defense response genes. The down‐regulation of defense pathway genes was observed in all Ws/L*er* Hybrid Mimics and one hybrid Mimic from the Col/L*er* system (CL_HM1), but not in CL_HM2, 3, and 4. Both small lines selected from the Col/L*er* system had defense genes up‐regulated with all five PR genes expressed at least twofold higher than the MPV. Genes in senescence pathways had expression patterns similar to the genes in defense response pathways pointing to an overlap between defense and senescence (Figure 6 and Figure S11). Some high yielding hybrids in Arabidopsis and crop species (stay‐green mutants) show delayed senescence which allows for more photosynthate production at the grain filling stage (Spano et al., 2003; Thomas & Howarth, 2000; You et al., 2007).

In Arabidopsis, we predict that we could generate high yielding Hybrid Mimics where a hybrid has a high level of hybrid vigor and where out‐crossing is excluded. Selections of hybrid Mimic‐like plants have been reported in a number of other species; bread wheat, field pea, and tomato have all been reported to have F5 – F6 lines with the same characteristics as the parental F1 hybrids and were stable in their properties in successive generations (Busch et al., 1971; Cregan & Busch, 1978; Sarawat et al., 1994; Williams, 1959). These lines are equivalent to Hybrid Mimics. The low number of generations needed to give rise to the true breeding high yielding lines in these crop species, just as in Arabidopsis, suggest that only a small number of loci making positive contributions to growth are responsible for the initiation of the Hybrid Mimics in each crop.

Data from three independent hybrids have shown a common change in the pattern of plant development important for the generation of hybrid vigor. Early germination of the hybrid results in early growth relative to parents and to a large final biomass. Hybrid Mimics have the same properties as the hybrid. Phenotypically, hybrids are larger than parents with larger leaves, thicker stems, and greater height (Birchler et al., 2003; Groszmann et al., 2014). Cell wall biosynthesis genes have increased expression in hybrids relative to parents.

In C24/L*er* hybrids and Hybrid Mimics, up‐regulation of the transcription factor PHYTOCHROME‐INTERACTING FACTOR 4 (PIF4) results in increased auxin biosynthesis and signaling. Several auxin‐responsive genes including cell expansion genes were up‐regulated in the F1 hybrids and hybrid mimics, suggesting that increased auxin biosynthesis and signaling contribute to the hybrid phenotype by promoting leaf growth (Wang, Liu, et al., 2018; Wang et al., 2017). Apart from the expression level of plant cell wall biosynthesis genes, the defense/growth balance is likely to be important. A reduction in the expression level of *PR* genes can lead to more energy being channeled to pathways which contribute to growth.

## ACCESSION NUMBERS

RNA‐seq data from this article are available in the GenBank database (accession no. GSE131682).

## CONFLICT OF INTEREST

The authors declare no conflict of interest.

## AUTHOR CONTRIBUTIONS

E.S.D, W.J.P, and L.W designed research; L.W and LM.W performed research; L.W and I.K.G analyzed the data; and L.W, E.S.D, and W.J.P wrote the paper.

## References

[pld3174-bib-0001] Ahn, J. W. , Verma, R. , Kim, M. , Lee, J. Y. , Kim, Y. K. , Bang, J. W. , … Pai, H. S. (2006). Depletion of UDP‐D‐apiose/UDP‐D‐xylose synthases results in rhamnogalacturonan‐II deficiency, cell wall thickening, and cell death in higher plants. Journal of Biological Chemistry, 281, 13708–13716. 16549428 10.1074/jbc.M512403200

[pld3174-bib-0002] Alonso‐Peral, M. M. , Trigueros, M. , Sherman, B. , Ying, H. , Taylor, J. M. , Peacock, W. J. , & Dennis, E. S. (2017). Patterns of gene expression in developing embryos of Arabidopsis hybrids. The Plant Journal, 89, 927–939. 10.1111/tpj.13432 27880012

[pld3174-bib-0003] Bechtold, U. , Lawson, T. , Mejia‐Carranza, J. , Meyer, R. C. , Brown, I. R. , Altmann, T. , … Mullineaux, P. M. (2010). Constitutive salicylic acid defences do not compromise seed yield, drought tolerance and water productivity in the Arabidopsis accession C24. Plant, Cell and Environment, 33, 1959–1973. 10.1111/j.1365-3040.2010.02198.x 20573051

[pld3174-bib-0004] Becnel, J. , Natarajan, M. , Kipp, A. , & Braam, J. (2006). Developmental expression patterns of Arabidopsis *XTH* genes reported by transgenes and genevestigator. Plant Molecular Biology, 61, 451–467. 16830179 10.1007/s11103-006-0021-z

[pld3174-bib-0005] Birchler, J. A. , Auger, D. L. , & Riddle, N. C. (2003). In search of the molecular basis of heterosis. The Plant Cell, 15, 2236–2239. 10.1105/tpc.151030 14523245 PMC540269

[pld3174-bib-0006] Boss, P. K. , Bastow, R. M. , Mylne, J. S. , & Dean, C. (2004). Multiple pathways in the decision to flower: Enabling, promoting, and resetting. The Plant Cell, 16, S18–S31. 10.1105/tpc.015958 15037730 PMC2643402

[pld3174-bib-0007] Busch, R. H. , Lucken, K. A. , & Frohberg, R. C. (1971). F_1_ hybrids versus random F_5_ line performance and estimates of genetic effects in spring wheat. Crop Science, 11, 357–361.

[pld3174-bib-0008] Cheng, S. H. , Zhuang, J. Y. , Fan, Y. Y. , Du, J. H. , & Cao, L. Y. (2007). Progress in research and development on hybrid rice: A super‐domesticate in China. Annals of Botany, 100, 959–966. 10.1093/aob/mcm121 17704538 PMC2759200

[pld3174-bib-0009] Cregan, P. B. , & Busch, R. H. (1978). Heterosis, inbreeding, and line performance in crosses of adapted spring wheats. Crop Science, 18, 247–251.

[pld3174-bib-0010] Crow, J. F. (1998). 90 years ago: The beginning of hybrid maize. Genetics, 148, 923–928. 9539413 10.1093/genetics/148.3.923 PMC1460037

[pld3174-bib-0011] Czechowski, T. , Stitt, M. , Altmann, T. , Udvardi, M. K. , & Scheible, W. R. (2005). Genome‐wide identification and testing of superior reference genes for transcript normalization in Arabidopsis. Plant Physiology, 39, 5–17. 10.1104/pp.105.063743 PMC1203353 16166256

[pld3174-bib-0012] Dan, Z. , Liu, P. , Huang, W. , Zhou, W. , Yao, G. , Hu, J. , … Zhu, Y. (2014). Balance between a higher degree of heterosis and increased reproductive isolation: A strategic design for breeding inter‐subspecific hybrid rice. PLoS ONE, 9, e93122 10.1371/journal.pone.0093122 24667442 PMC3965518

[pld3174-bib-0013] Denance, N. , Sanchez‐Vallet, A. , Goffner, D. , & Molina, A. (2013). Disease resistance or growth: The role of plant hormones in balancing immune responses and fitness costs. Frontiers in Plant Science, 4, 155 10.3389/fpls.2013.00155 23745126 PMC3662895

[pld3174-bib-0014] Endler, A. , & Persson, S. (2011). Cellulose synthases and synthesis in Arabidopsis. Molecular Plant, 4, 199–211. 21307367 10.1093/mp/ssq079

[pld3174-bib-0015] Eulgem, T. , Rushton, P. J. , Robatzek, S. , & Somssich, I. E. (2000). The WRKY superfamily of plant transcription factors. Trends in Plant Science, 5, 199–206. 10.1016/S1360-1385(00)01600-9 10785665

[pld3174-bib-0016] Fujimoto, R. , Taylor, J. M. , Shirasawa, S. , Peacock, W. J. , & Dennis, E. S. (2012). Heterosis of Arabidopsis hybrids between C24 and Col is associated with increased photosynthesis capacity. Proceedings of the National Academy of Sciences of the United States of America, 109, 7109–7114. 10.1073/pnas.1204464109 22493265 PMC3344962

[pld3174-bib-0017] Glowacki, S. , Macioszek, V. K. , & Kononowicz, A. K. (2011). R proteins as fundamentals of plant innate immunity. Cellular & Molecular Biology Letters, 16, 1–24. 20585889 10.2478/s11658-010-0024-2 PMC6275759

[pld3174-bib-0018] Gonzalez‐Bayon, R. , Shen, Y. , Groszmann, M. , Zhu, A. , Wang, A. , Allu, A. D. , … Greaves, I. K. (2019). Senescence and defense pathways contribute to heterosis. Plant Physiology, 180, 240–254. 10.1104/pp.18.01205 30710054 PMC6501064

[pld3174-bib-0019] Greaves, I. K. , Gonzalez‐Bayon, R. , Wang, L. , Zhu, A. , Liu, P. C. , Groszmann, M. , … Dennis, E. S. (2015). Epigenetic changes in hybrids. Plant Physiology, 168, 1197–1205. 10.1104/pp.15.00231 26002907 PMC4528738

[pld3174-bib-0020] Groszmann, M. , Gonzalez‐Bayon, R. , Greaves, I. K. , Wang, L. , Huen, A. K. , Peacock, W. J. , & Dennis, E. S. (2014). Intraspecific Arabidopsis hybrids show different patterns of heterosis despite the close relatedness of the parental genomes. Plant Physiology, 166, 265–280. 10.1104/pp.114.243998 25073707 PMC4149712

[pld3174-bib-0021] Groszmann, M. , Gonzalez‐Bayon, R. , Lyons, R. L. , Greaves, I. K. , Kazan, K. , Peacock, W. J. , & Dennis, E. S. (2015). Hormone‐regulated defense and stress response networks contribute to heterosis in Arabidopsis F1 hybrids. Proceedings of the National Academy of Sciences of the United States of America, 112, E6397–E6406. 26527659 10.1073/pnas.1519926112 PMC4655576

[pld3174-bib-0022] Hu, H. , Zhang, R. , Feng, S. , Wang, Y. , Wang, Y. , Fan, C. , … Peng, L. C. (2018). Three AtCesA6‐like members enhance biomass production by distinctively promoting cell growth in Arabidopsis. Plant Biotechnology Journal, 16, 976–988. 28944540 10.1111/pbi.12842 PMC5902768

[pld3174-bib-0023] Jung, C. , Pillen, K. , Staiger, D. , Coupland, G. , & von Korff, M. (2017). Editorial: Recent advances in flowering time control. Frontiers in Plant Science, 7, 2011 10.3389/fpls.2016.02011 28105041 PMC5214091

[pld3174-bib-0024] Klinghammer, M. , & Tenhaken, R. (2007). Genome‐wide analysis of the UDP‐glucose dehydrogenase gene family in Arabidopsis, a key enzyme for matrix polysaccharides in cell walls. Journal of Experimental Botany, 58, 3609–3621. 10.1093/jxb/erm209 18057039

[pld3174-bib-0025] Li, H. , Yang, Q. , Fan, N. , Zhang, M. , Zhai, H. , Ni, Z. , & Zhang, Y. (2017). Quantitative trait locus analysis of heterosis for plant height and ear height in an elite maize hybrid zhengdan 958 by design III. BMC Genetics, 18, 36 10.1186/s12863-017-0503-9 28415964 PMC5392948

[pld3174-bib-0026] Li, N. N. , Chen, L. , Li, X. H. , Li, Q. , Zhang, W. B. , Takechi, K. , … Lin, X. F. (2017). Overexpression of UDP‐glucose dehydrogenase from *Larix gmelinii* enhances growth and cold tolerance in transgenic *Arabidopsis thaliana* . Biologia Plantarum, 61, 95–105. 10.1007/s10535-016-0657-8

[pld3174-bib-0027] Love, M. I. , Huber, W. , & Anders, S. (2014). Moderated estimation of fold change and dispersion for RNA‐seq data with DESeq2. Genome Biology, 15, 550 10.1186/s13059-014-0550-8 25516281 PMC4302049

[pld3174-bib-0028] McFarlane, H. E. , Doring, A. , & Persson, S. (2014). The cell biology of cellulose synthesis. Annual Review of Plant Biology, 65, 69–94. 10.1146/annurev-arplant-050213-040240 24579997

[pld3174-bib-0029] Meyer, R. C. , Torjek, O. , Becher, M. , & Altmann, T. (2004). Heterosis of biomass production in Arabidopsis establishment during early development. Plant Physiology, 134, 1813–1823. 10.1104/pp.103.033001 15064384 PMC419853

[pld3174-bib-0030] Meyer, R. C. , Witucka‐Wall, H. , Becher, M. , Blacha, A. , Boudichevskaia, A. , Dormann, P. , … Altmann, T. (2012). Heterosis manifestation during early Arabidopsis seedling development is characterized by intermediate gene expression and enhanced metabolic activity in the hybrids. The Plant Journal, 71, 669–683. 10.1111/j.1365-313X.2012.05021.x 22487254

[pld3174-bib-0031] Miller, M. , Song, Q. , Shi, X. , Juenger, T. E. , & Chen, Z. J. (2015). Natural variation in timing of stress‐responsive gene expression predicts heterosis in intraspecific hybrids of Arabidopsis. Nature Communications, 6, 7453 10.1038/ncomms8453 26154604

[pld3174-bib-0032] Mishina, T. E. , & Zeier, J. (2006). The Arabidopsis flavin‐dependent monooxygenase FMO1 is an essential component of biologically induced systemic acquired resistance. Plant Physiology, 141, 1666–1675. 10.1104/pp.106.081257 16778014 PMC1533925

[pld3174-bib-0033] Pajerowska‐Mukhtar, K. M. , Wang, W. , Tada, Y. , Oka, N. , Tucker, C. L. , Fonseca, J. P. , & Dong, X. (2012). The HSF‐like transcription factor TBF1 is a major molecular switch for plant growth‐to‐defense transition. Current Biology, 22, 103–112. 22244999 10.1016/j.cub.2011.12.015 PMC3298764

[pld3174-bib-0034] Reboul, R. , Geserick, C. , Pabst, M. , Frey, B. , Wittmann, D. , Lutz‐Meindl, U. , … Tenhaken, R. (2011). Down‐regulation of UDP‐glucuronic acid biosynthesis leads to swollen plant cell walls and severe developmental defects associated with changes in pectic polysaccharides. Journal of Biological Chemistry, 286, 39982–39992. 21949134 10.1074/jbc.M111.255695 PMC3220558

[pld3174-bib-0035] Rose, J. K. C. , Braam, J. , Fry, S. C. , & Nishitani, K. (2002). The XTH family of enzymes involved in xyloglucan endotransglucosylation and endohydrolysis: Current perspectives and a new unifying nomenclature. Plant and Cell Physiology, 43, 1421–1435. 10.1093/pcp/pcf171 12514239

[pld3174-bib-0036] Saeki, N. , Kawanabe, T. , Ying, H. , Shimizu, M. , Kojima, M. , Abe, H. , … Fujimoto, R. (2016). Molecular and cellular characteristics of hybrid vigour in a commercial hybrid of Chinese cabbage. BMC Plant Biology, 16, 45 10.1186/s12870-016-0734-3 26882898 PMC4756405

[pld3174-bib-0037] Sarawat, P. , Stoddard, F. L. , & Marshall, D. R. (1994). Derivation of superior F5 lines from heterotic hybrids in pea. Euphytica, 73, 265–272. 10.1007/BF00036705

[pld3174-bib-0038] Sarkissian, I. V. , Harris, W. , & Kessinger, M. A. (1964). Differential rates of development of heterotic + nonheterotic young maize seedlings. i. correlation of differential morphological development with physiological differences in germinating seeds. Proceedings of the National Academy of Sciences of the United States of America, 51, 212–218. 16591144 10.1073/pnas.51.2.212 PMC300051

[pld3174-bib-0039] Simpson, G. G. , & Dean, C. (2002). Flowering ‐ Arabidopsis, the rosetta stone of flowering time? Science, 296, 285–289. 10.1126/science.296.5566.285 11951029

[pld3174-bib-0040] Song, Q. , Ando, A. , Xu, D. , Fang, L. , Zhang, T. , Huq, E. , … Chen, Z. J. (2018). Diurnal down‐regulation of ethylene biosynthesis mediates biomass heterosis. Proceedings of the National Academy of Sciences of the United States of America, 115, 5606–5611. 10.1073/pnas.1722068115 29735680 PMC6003462

[pld3174-bib-0041] Spano, G. , Di Fonzo, N. , Perrotta, C. , Platani, C. , Ronga, G. , Lawlor, D. W. , … Shewry, P. R. (2003). Physiological characterization of 'stay green' mutants in durum wheat. Journal of Experimental Botany, 54, 1415–1420. 12709488 10.1093/jxb/erg150

[pld3174-bib-0042] Thomas, H. , & Howarth, C. J. (2000). Five ways to stay green. Journal of Experimental Botany, 51, 329–337. 10938840 10.1093/jexbot/51.suppl_1.329

[pld3174-bib-0043] Thompson, D. S. (2005). How do cell walls regulate plant growth? Journal of Experimental Botany, 56, 2275–2285. 16061505 10.1093/jxb/eri247

[pld3174-bib-0044] Tian, T. , Liu, Y. , Yan, H. , You, Q. , Yi, X. , Du, Z. , … Su, Z. (2017). agriGO v2.0: A GO analysis toolkit for the agricultural community, 2017 update. Nucleic Acids Research, 45, W122–W129. 28472432 10.1093/nar/gkx382 PMC5793732

[pld3174-bib-0045] Wang, L. , Greaves, I. K. , Groszmann, M. , Wu, L. M. , Dennis, E. S. , & Peacock, W. J. (2015). Hybrid mimics and hybrid vigor in Arabidopsis. Proceedings of the National Academy of Sciences of the United States of America, 112, E4959–E4967. 26283378 10.1073/pnas.1514190112 PMC4568211

[pld3174-bib-0046] Wang, L. , Liu, P. C. , Wu, L. M. , Tan, J. , Peacock, W. J. , & Dennis, E. S. (2018). Cotyledons contribute to plant growth and hybrid vigor in Arabidopsis. Planta, 249, 1107–1118. 10.1007/s00425-018-3068-6 30552582

[pld3174-bib-0047] Wang, L. , Wu, L. M. , Greaves, I. K. , Zhu, A. , Dennis, E. S. , & Peacock, W. J. (2017). PIF4‐controlled auxin pathway contributes to hybrid vigor in *Arabidopsis thaliana* . Proceedings of the National Academy of Sciences of the United States of America, 114, E3555–E3562. 28396418 10.1073/pnas.1703179114 PMC5410812

[pld3174-bib-0048] Wang, Y. , Schuck, S. , Wu, J. , Yang, P. , Doring, A. C. , Zeier, J. , & Tsuda, K. (2018). A MPK3/6‐WRKY33‐ALD1‐pipecolic acid regulatory loop contributes to systemic acquired resistance. The Plant Cell, 30, 2480–2494. 10.1105/tpc.18.00547 30228125 PMC6241261

[pld3174-bib-0049] Williams, W. (1959). The isolation of 'pure lines' from F1 hybrids of tomato and the problem of heterosis in inbreeding crop species. Journal of Agricultural Science, 53, 347–353.

[pld3174-bib-0050] Xu, J. , Meng, J. , Meng, X. , Zhao, Y. , Liu, J. , Sun, T. , … Zhang, S. (2016). Pathogen‐Responsive MPK3 and MPK6 reprogram the biosynthesis of indole glucosinolates and their derivatives in Arabidopsis immunity. The Plant Cell, 28, 1144–1162. 10.1105/tpc.15.00871 27081184 PMC4904669

[pld3174-bib-0051] You, S. C. , Cho, S. H. , Zhang, H. , Paik, H. C. , Lee, C. H. , Li, J. , … Paek, N. C. (2007). Quantitative trait loci associated with functional stay‐green SNU‐SG1 in rice. Molecules and Cells, 24, 83–94. 17846502

[pld3174-bib-0052] Zablackis, E. , Huang, J. , Muller, B. , Darvill, A. G. , & Albersheim, P. (1995). Structure of plant‐cell walls. 34. characterization of the cell‐wall polysaccharides of *Arabidopsis thaliana* leaves. Plant Physiology, 107, 1129–1138. 10.1104/pp.107.4.1129 7770522 PMC157245

[pld3174-bib-0053] Zhu, A. , Greaves, I. K. , Liu, P. C. , Wu, L. M. , Dennis, E. S. , & Peacock, W. J. (2016). Early changes of gene activity in developing seedlings of Arabidopsis hybrids relative to parents may contribute to hybrid vigour. The Plant Journal, 88, 597–607. 10.1111/tpj.13285 27460790

