# Supplementary material for: In Arabidopsis hybrids and Hybrid Mimics, up‐regulation of cell wall biogenesis is associated with the increased plant size
Source: Plant Direct. 2019 Nov 6;3(11):e00174. doi: 10.1002/pld3.174 (PMC6834268; doi:10.1002/pld3.174)
Supplement: Supplementary file 1 [file PLD3-3-e00174-s001.pdf]

Fig.S1

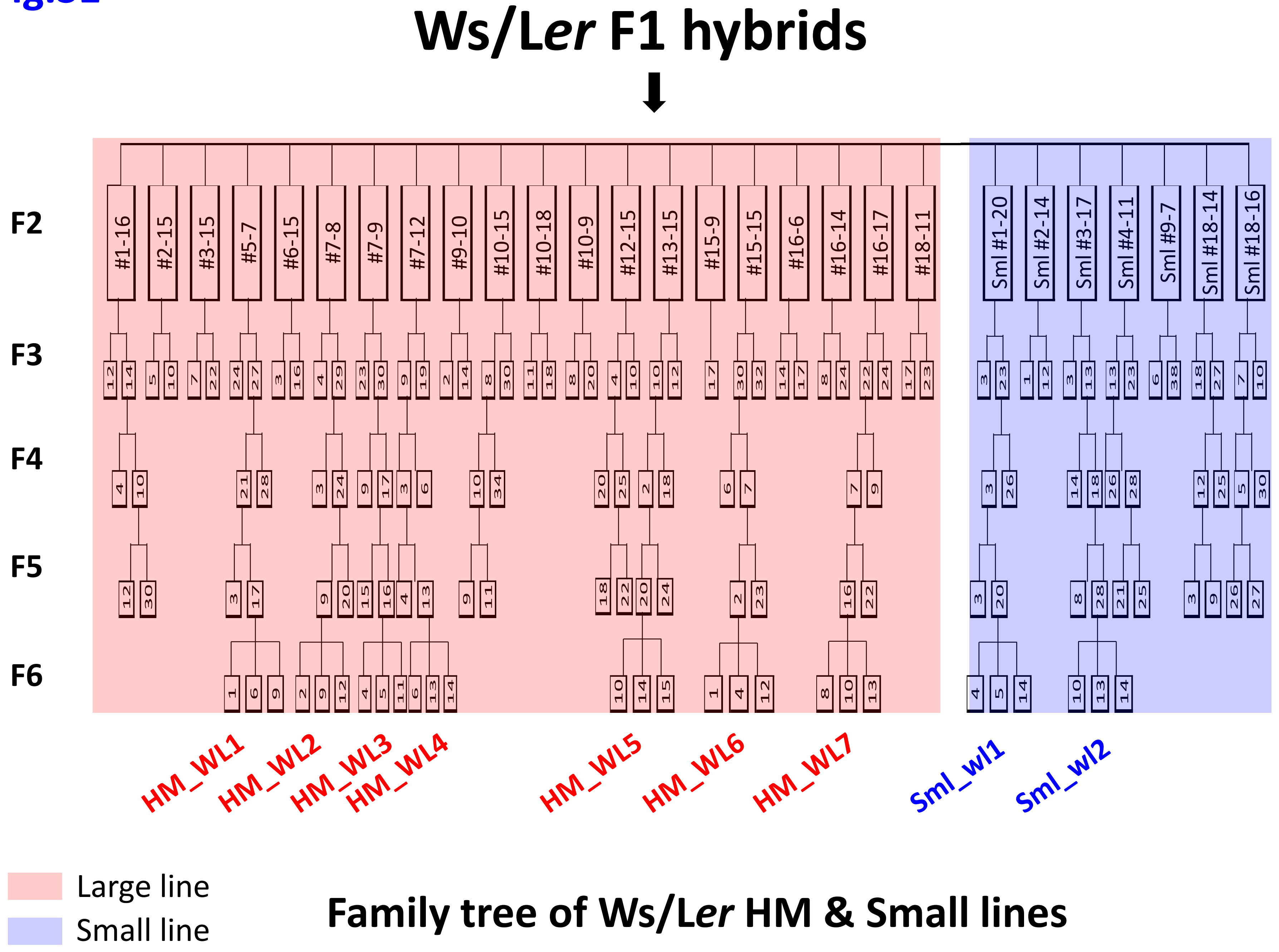

Family tree of Ws/*Ler* HM & Small lines

Figure S1. The genealogical tree of Hybrid Mimic selection from Ws/L*er* system. 20 large and 7 small F2 plants were selected from a F2 population of 300 plants. The 20 large and 7 small F2 plants were selfed and 30 F3 seeds from each grown as F3 lines. 39 large and 14 small F3 plants were selected to produce F4 offspring. In F4, 20 large and 10 small plant lines were analysed with 30 plants grown per line, and 20 large and 10 small F4 plants were selected for generating the F5; seven large plants and two small plants were selected in the F5 to produce the F6 seeds. The seven F6 large plant lines were referred as Ws/L*er* Hybrid Mimic 1-7 (WL_HM1-7). The two F6 small lines were referred as Ws/L*er* small line 1 and 2 (wl_sml1-2).

Fig.S2

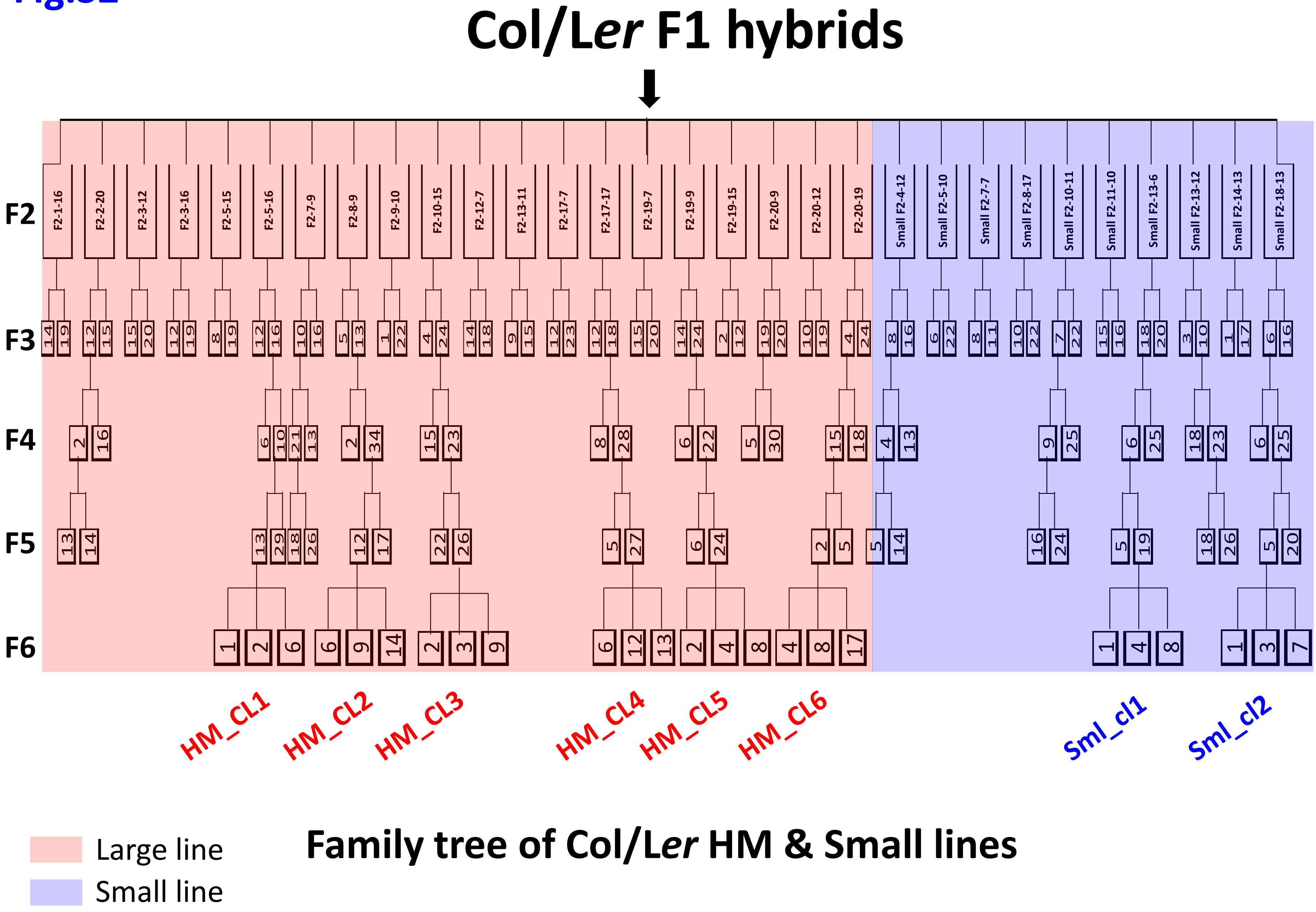

Family tree of Col/*Ler* HM & Small lines

Figure S2. The genealogical tree of Hybrid Mimic selection from Col/L*er* system. 20 large and 10 small F2 plants were selected from a F2 population of 300 plants. The 20 large and 10 small F2 plants were selfed and 30 F3 seeds from each grown as F3 lines. 40 large and 20 small F3 plants were selected to produce F4 offspring. In F4, 20 large and 10 small plant lines were analysed with 30 plants grown per line, and 18 F4 large and 10 small plants were selected for generating F5; 6 large plants and 2 small plants were selected in the F5 to produce the F6 seeds. The selected six F6 large plant lines were referred as Col/L*er* Hybrid Mimic 1-6 (CL_HM1-6). The selected two F6 small lines were referred as Col/L*er* small line 1 and 2 (cl_sml1-2). CL_HM5 and CL_HM6 were excluded from the transcriptome analysis due to unsatisfactory phenotypes of plant size at sampling day (25 DAS).

Fig.S3

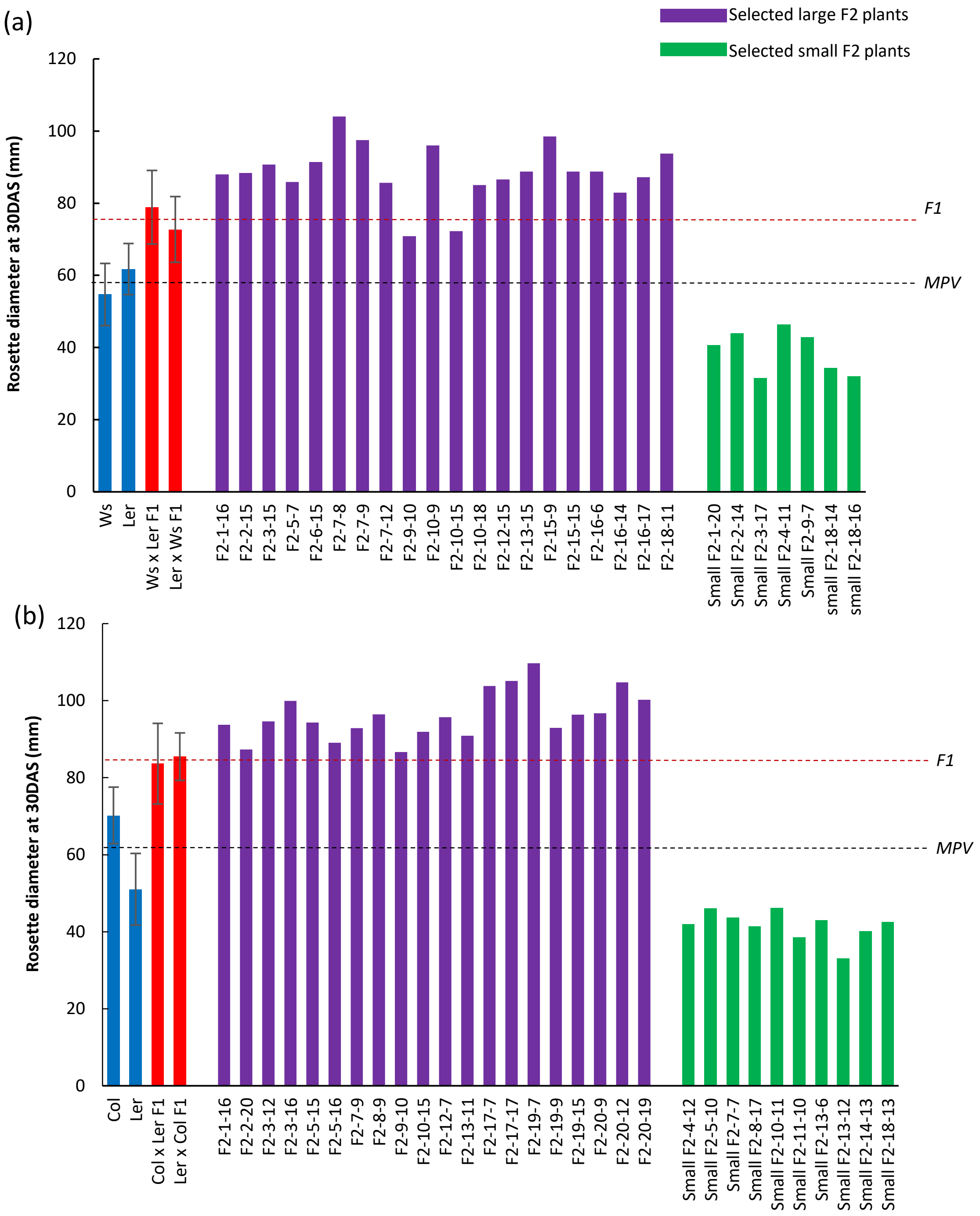

(a)
Selected large F2 plants
Selected small F2 plants
Rosette diameter at 30DAS (mm)
0
20
40
60
80
100
120
F1
MPV
Ws
Ler
Ws x Ler F1
Ler x Ws F1
F2-1-16
F2-2-15
F2-3-15
F2-5-7
F2-6-15
F2-7-8
F2-7-9
F2-7-12
F2-9-10
F2-10-9
F2-10-15
F2-10-18
F2-12-15
F2-13-15
F2-15-9
F2-15-15
F2-16-6
F2-16-14
F2-16-17
F2-18-11
Small F2-1-20
Small F2-2-14
Small F2-3-17
Small F2-4-11
Small F2-9-7
small F2-18-14
small F2-18-16
(b)
Rosette diameter at 30DAS (mm)
0
20
40
60
80
100
120
F1
MPV
Col
Ler
Col x Ler F1
Ler x Col F1
F2-1-16
F2-2-20
F2-3-12
F2-3-16
F2-5-15
F2-5-16
F2-7-9
F2-8-9
F2-9-10
F2-10-15
F2-12-7
F2-13-11
F2-17-7
F2-17-17
F2-19-7
F2-19-9
F2-19-15
F2-20-9
F2-20-12
F2-20-19
Small F2-4-12
Small F2-5-10
Small F2-7-7
Small F2-8-17
Small F2-10-11
Small F2-11-10
Small F2-13-6
Small F2-13-12
Small F2-14-13
Small F2-18-13

Figure S3. Rosette diameters of large and small F2 plants selected from Ws/L*er* and Col/L*er* systems at 30 DAS. (a) 20 large and 7 small F2 plants were selected from Ws/L*er* system. (b) 20 large and 10 small F2 plants were selected from Col/L*er* system. The blue/red/purple/green colours represent parents/hybrids/large F2 plants/small F2 plants. Black dotted line represents MPV. Red dotted line represents the average rosette diameter of two reciprocal hybrids. Error bars = SE. n > 20.

Fig.S4

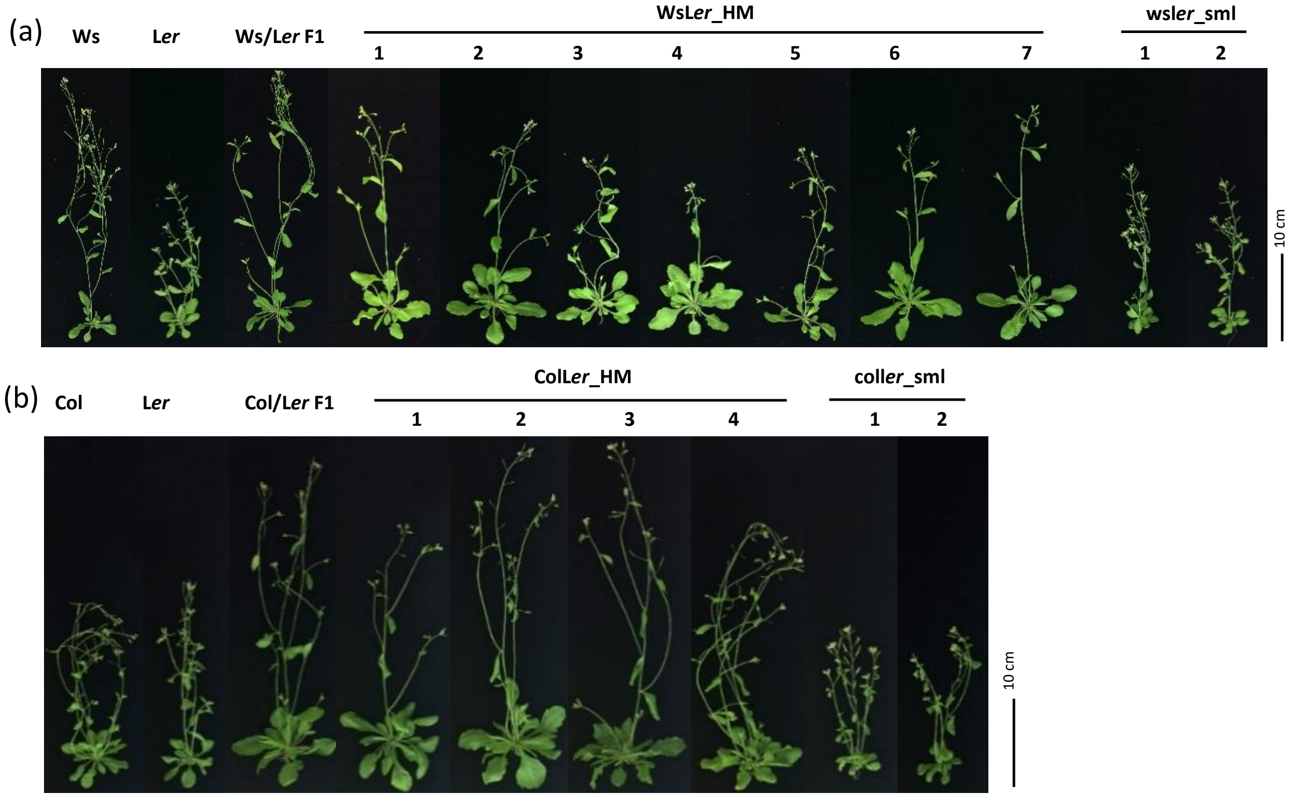

Figure S4. Hybrid Mimics selected from Ws/L*er* and Col/L*er* systems had increased rosette sizes at 30 DAS. (a) Plant phenotypes of the wild-type parents Ws and L*er*, Ws/L*er* hybrids, representative plants of seven Hybrid Mimic lines (WsL*er*_HM 1-7) and two small lines (wsl*er*_sml1 and 2) at 30 DAS. (b) Plant phenotypes of the parents Col and L*er*, Col/L*er* hybrids, representative plants of four Hybrid Mimic lines (ColL*er*_HM 1-4) and two small line (coll*er*_sml1 and 2) at 30 DAS. Scale bar = 10 cm.

Fig.S5

(a)

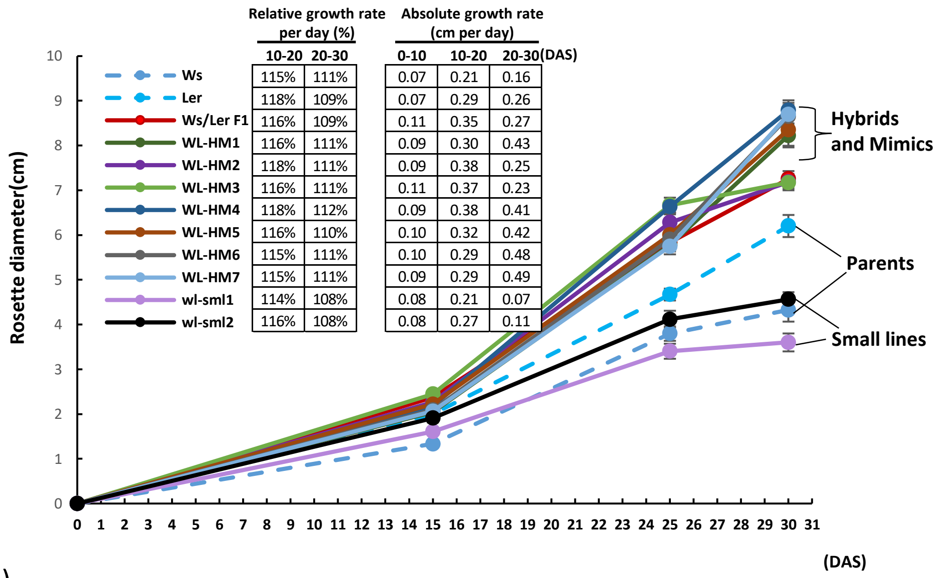

(b)

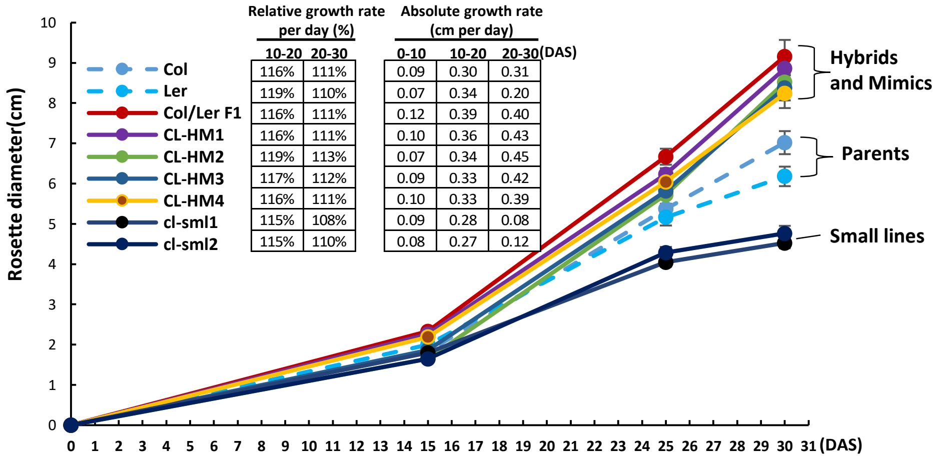

(c)

	Ws	*Ler*	Ws/Ler F1	WL_HM1	WL_HM2	WL_HM3	WL_HM4	WL_HM5	WL_HM6	WL_HM7	wl_sml1	wl_sml2
**RD at 15 DAS (cm)**	1.3	2.0	2.4	2.0	2.3	2.4	2.2	2.2	2.1	2.1	1.6	1.9
**SE**	0.05	0.08	0.08	0.08	0.06	0.06	0.04	0.06	0.06	0.06	0.06	0.10

	Col	*Ler*	Col/Ler F1	CL_HM1	CL_HM2	CL_HM3	CL_HM4	cl_sml1	cl_sml2
**RD at 15 DAS (cm)**	1.8	2.0	2.3	2.3	1.7	1.9	2.2	1.8	1.6
**SE**	0.03	0.08	0.07	0.03	0.05	0.07	0.06	0.05	0.06

Figure S5. Hybrid Mimics selected from Ws/L*er* and Col/L*er* systems showed growth patterns similar to the hybrids. (a) Growth course of parents Ws and L*er*, Ws/L*er* hybrids, seven Hybrid Mimic lines (WsL*er*_HM 1-7) and two small lines (wsl*er*_sml1-2). Error bars = SE, n = 12 - 15. (b) Growth course of parents Col and L*er*, Col/L*er* hybrids, four Hybrid Mimic lines (ColL*er*_HM 1 and 4) and two small lines (coll*er*_sml1-2). Error bars = SE, n = 17 - 20. (c) The rosette diameter of parents, hybrids and F7 lines in the Ws/L*er* and Col/L*er* systems.

Fig.S6

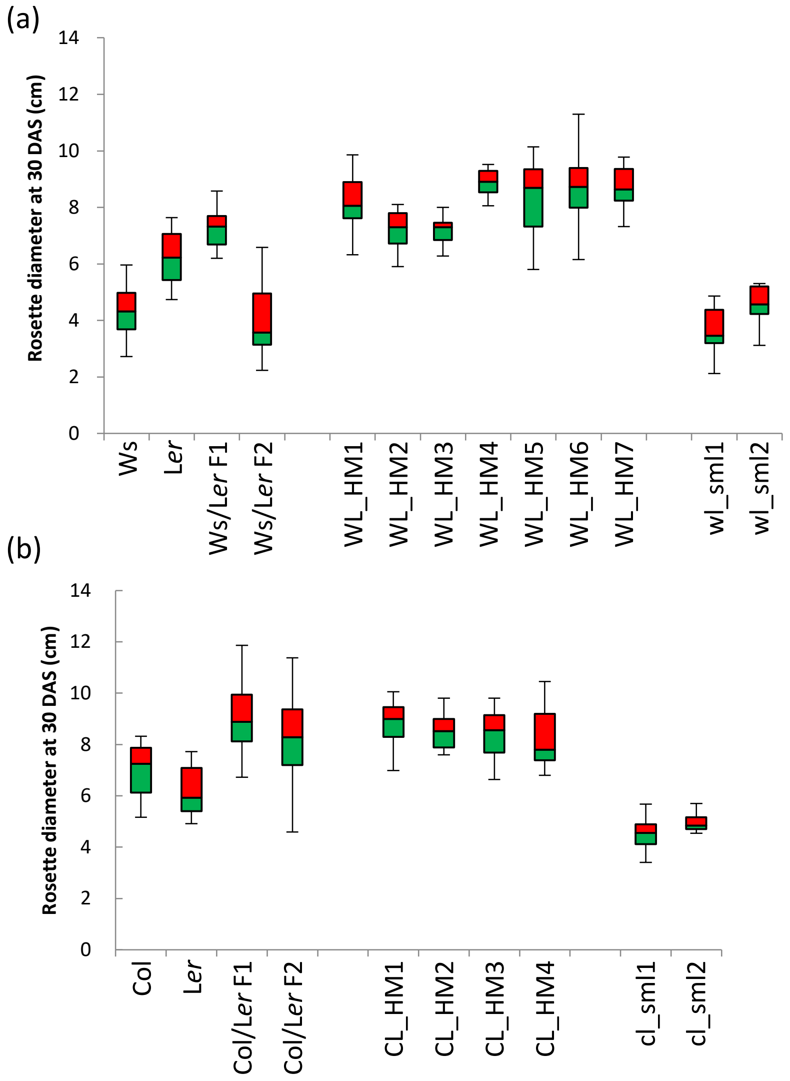

Figure S6. Hybrid Mimics showed large rosette sizes and uniformity close to F1. (a) Box-plots showing the uniformity of parent Ws and L*er*, Ws/L*er* hybrids, F2 population, seven F7 Hybrid Mimic lines (WsL*er*_HM 1-7) and two F7 small line (wsl*er*_sml1-2) in rosette diameter at 30 DAS. Error bars = SE, n = 14 - 20. (b) Box-plots showing the uniformity of parent Col and L*er*, Col/L*er* hybrids, F2 population, four F7 Hybrid Mimic lines (ColL*er*_HM 1 and 4) and two F7 small line (coll*er*_sml1-2) in rosette diameter at 30 DAS. Error bars = SE, n = 11 - 20.

Fig.S7

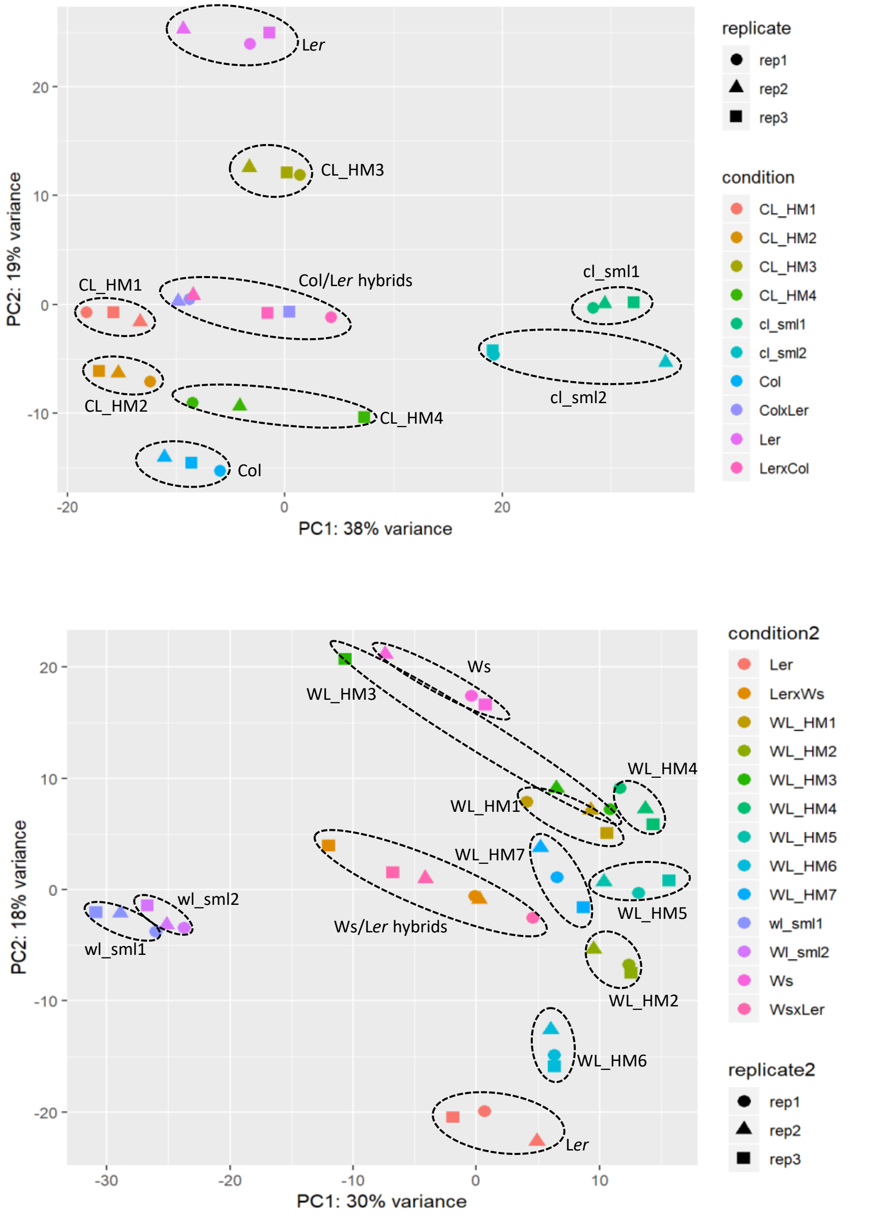

Figure S7. Principal component analysis (PCA) of the transcriptome data showed similarity of the gene expression patterns in hybrids and Mimics.

Fig.S8

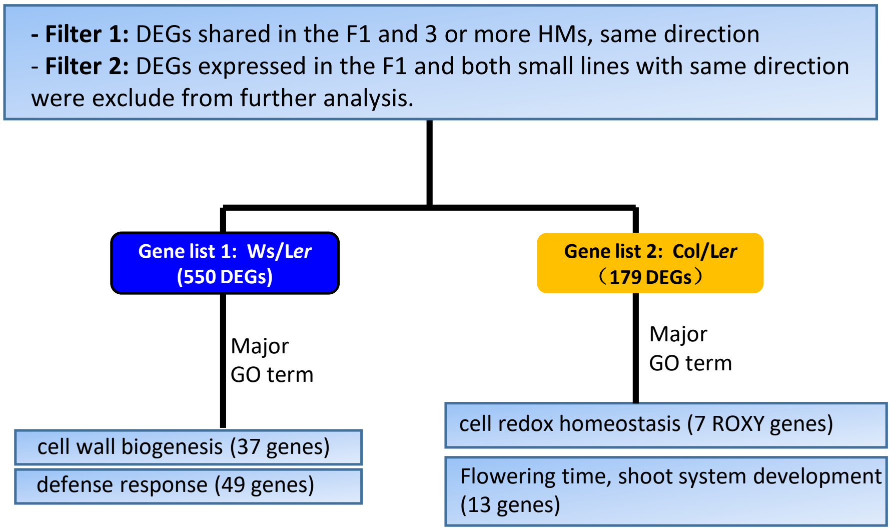

Figure S8. Workflow of identification of major pathways in the shared differentially expression genes in the F1 hybrids and Hybrid Mimics.

Fig.S9

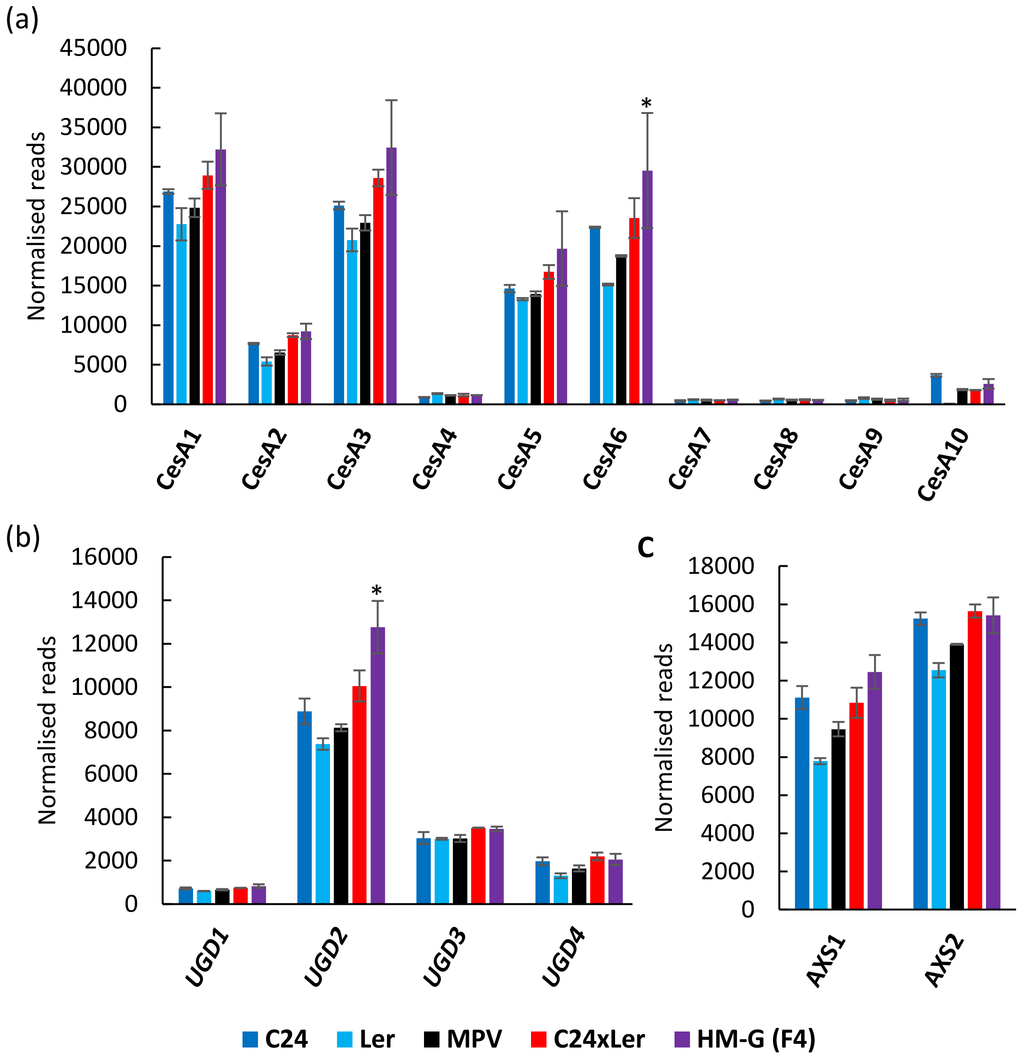

Figure S9. Cell wall biosynthesis genes were up-regulated in the C24/L*er* hybrids and Hybrid Mimic line. Relative expression of 10 *CesA* genes (*CesA1-10*) (a), four *UGD* genes (*UGD1-4*) (b) and two *AXS* genes (*AXS1* and *AXS2*) (C) in the rosette leaves of 28 d-old parents C24 and L*er*, C24xL*er* hybrid, F4 Hybrid Mimic line HM-G. For each gene the expression levels in each plant line were normalized reads. The data represents the mean of two biological replicates. Error bars = SE. * indicates significant differences at *P* (Student's *t* test) < 0.05 from MPV.

Fig.S10

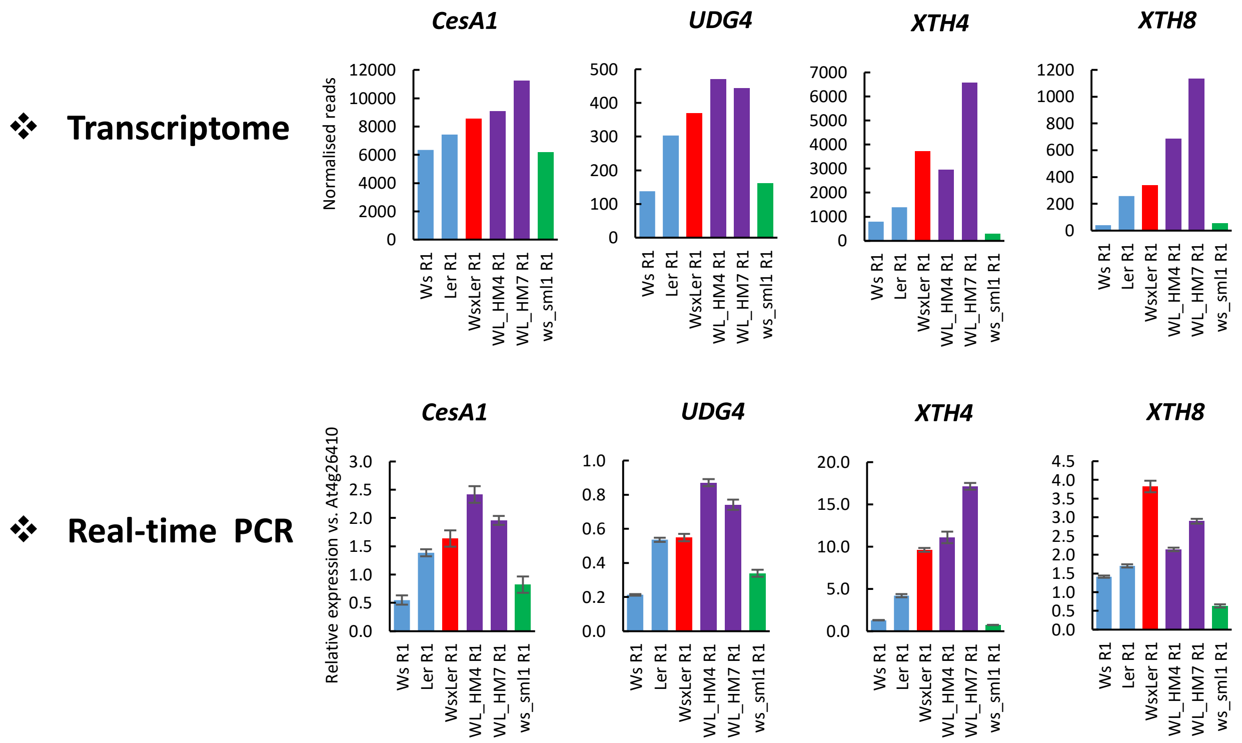

Figure S10. The up-regulation of four cell wall related genes in Ws/L*er* Hybrid and Mimics was validated by quantitative real-time PCR. Error bars = SE of three technical replicates.

Fig.S11

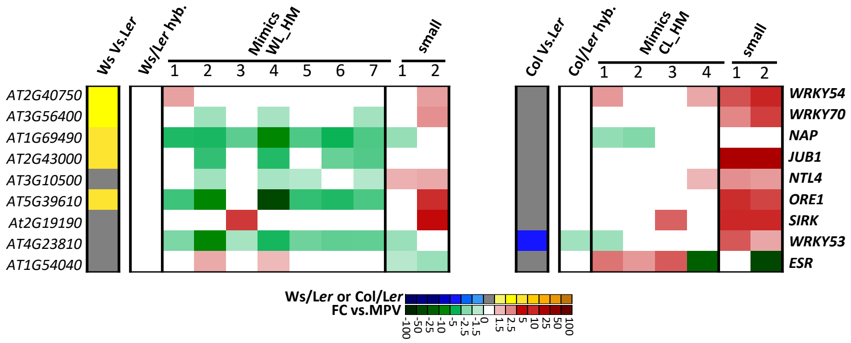

Figure S11. The expression levels of genes associated with senescence in the parents, hybrids and F7 lines. Different red/green colors indicate the up-/down-regulated fold change from the MPV. Blue/yellow colour indicates the comparison between the two parents in Ws/L*er* or Col/L*er* system.

Fig.S12

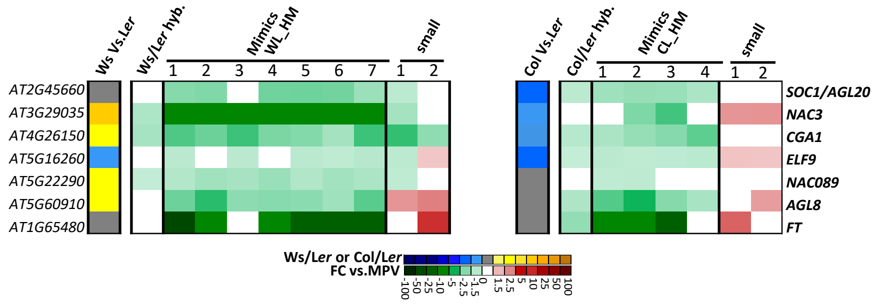

Figure S12. Hybrids and Mimics had differentially expressed genes associated with flowering time and shoot system development. Different red/green colors indicate the up-/down-regulated fold change from the MPV. Blue/yellow colour indicates the comparison between the two parents in Ws/L*er* or Col/L*er* system.

Fig.S13

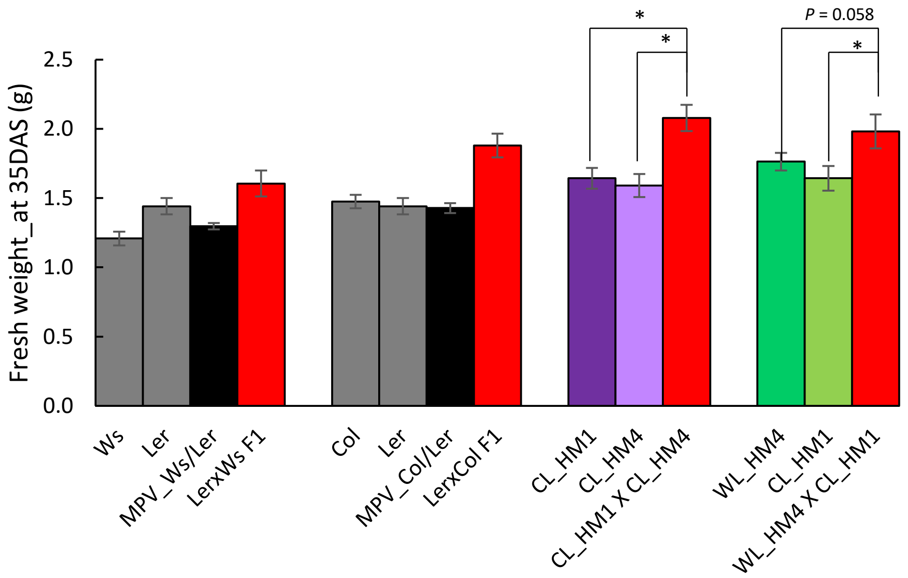

Figure S13. Fresh weights of wild type parents Ws, Col and L*er*, L*er*xWs F1 hybrids, L*er*xWs F1 hybrids, Hybrid Mimics and inter cross progeny at 35 DAS. Error bars = SE. n > 10. *indicates significant differences at *P* (Student's t test) < 0.05.
