# Supplementary material for: In Arabidopsis hybrids and Hybrid Mimics, up‐regulation of cell wall biogenesis is associated with the increased plant size
Source: Plant Direct. 2019 Nov 6;3(11):e00174. doi: 10.1002/pld3.174 (PMC6834268; doi:10.1002/pld3.174)
Supplement: Supplementary file 3 [file PLD3-3-e00174-s003.pdf]

Respond to reviewers

Replies to reviewer's comments are in blue letters.

REVIEWER COMMENTS:

Reviewer: 1

The genetic basis of heterosis is a widely interesting topic and has been explored extensively for a long time. Many studies have made genome-wide comparison of gene expression between hybrids and their parents, and have identified some genes and biological pathways potential contributing to heterosis for biomass and yield in Arabidopsis and crops. In the manuscript of "In Arabidopsis hybrids and Hybrid Mimics up-regulation of cell wall biogenesis is associated with increased plant size" by Wang et al., the authors generating F5/F6 Hybrid Mimics from two hybrid crosses in Arabidopsis. Transcriptome analysis showed that cell wall biosynthesis and cell wall expansion genes were unregulated in hybrids and Hybrid Mimics compared to parents. By using a different experiment design from most other studies, this study may provide new clue for our understand of molecular basis of heterosis.

Q1: The major concern about the study is that the authors should make experimental validation of the upregulation of cell wall biogenesis in hybrid and Hybrid Mimics using real-time quantitative PCR.

Re: The up-regulation of four cell wall related genes in Ws/L*er* Hybrid and Mimics was validated by quantitative real-time PCR. Data is provided in Figure S10.

Q2: Moreover, some important stuides on biomass heterosis in Arabidopsis published in recent years should not be ignored in thie manuscript.

Re: A paragraph has been added in the introduction section regarding important studies on biomass heterosis in Arabidopsis published recently. Please see page 3, lines 11-21.

Reviewer: 2

In this study, Wang and co-workers establish homozygous hybrid populations between different Arabidopsis ecotypes. By selecting progeny based on phenotypes, they show that these lines can mimic the hybrid vigour typically observed in the F1 of similar crosses. They then move forward to ask what gene expression networks may contribute to the observed phenotypes and undertake several RNA sequencing analyses. Finally, they provide evidence that new crosses between the homozygous hybrids lead to even further hybrid vigour in the following F1 population.

major points:

The authors have had multiple paper where they outline the homozygous populations that mimic the hybrid vigour of the F1 in terms of phenotype, freshweight etc (see for example Wang et al., 2015; 2017). Hence, this part of the paper is therefore rather archival and the main point of this paper would then be the comparative expression analyses done via RNA seq. Many thousands of genes are shown to be differentially expressed between the different hybrids, parent plants and hybrids that are smaller than the parent lines. While many of the genes make sense in context of what we understand of growth and plant biology, due to the sheer amount of genes they are unlikely to increase our molecular understanding of hybrid vigour. The paper is therefore rather a descriptive work with very little insights into what may be driving the growth changes.

Q3: It would of course also be nice to show that the expression of some of the genes really are up-regulated via for example qPCR as an independent way to assess expression.

Re: The up-regulation of four cell wall related genes in Ws/L*er* Hybrid and Mimics was validated by quantitative real-time PCR. Data is provided in Figure S10.

Q4: It is unclear to me how much of the improved performance is linked to growth conditions of the homozygous mimic progeny? Are they also performing better under other growth conditions than the standard conditions outlined in the paper?

Re: We have not tested the performance of these Hybrid Mimics under different growth conditions. We understand that the plant phenotypes are associated with how its genetics and epigenetics properties respond to the growth environment. Hybrids show different growth performances or have different levels of Hybrid vigor under different growth conditions. It is possible that Hybrid Mimics could also have different phenotypes under other growth conditions.

Q5: In terms of the writing; the introduction contains quite some results; I would suggest the authors to remove this and instead give a broader background to the processes they are later referring to with their expression analyse and how these processes affect growth. The result section is largely consisting of the RNA seq analyses. This could easily be merged with the discussion part to avoid repletion in the discussion part.

Re: Manuscript has been revised as the review's suggestion. Some sentences which describe results have been removed. A broader background regarding recent findings of hybrid vigor has been added. Please see the introduction section. Please see page 3, lines 11-21.

Q6: While I recognize that some aspects of biology are directed by gene expression, why did the authors not choose proteomics or post-translational modifications of proteins to at least get closer to the execution step?

Re: The identification of the altered biological pathways in the hybrids and Mimics comes from transcriptome data. We do not have data in protein levels.

minor points:

Q7: Figure 1; I suggest the authors to use separate bars for the FW for shoots and roots as it would be easier to directly compare the different genotypes.

Re: Modifications have been made as review's suggestion. Please see Figure 1.

Reviewer: 3

General Comments:

Q8: The authors often declare similarities or differences without presenting the statistical analyses underpinning these results.

Re: Threshold for the significant difference (*P* < 0.05) between two items was indicated in the text at multiple places. Eg. Page 6 lines 2, 8, 19.

Q9: Molecular analyses took place at a late time point (25 DAS). Are the findings the cause or the consequence of heterosis?

Re: Our data indicated that several genes involved in cell wall biosynthesis such as *CELLULOSE SYNTHASE (CesA)* genes were upregulated in the the F1 hybrids and Hybrid Mimics. Considering the results published by Hu et al (2018) that transgenic plants overexpressing CesA2, 5 or 6 were taller than the wild-type and produced 20% more biomass in 7-week-old mature plants, we conclude that our findings of up-regulation of cell wall biosynthesis genes in the hybrids and Mimics are causes of heterosis.

Q10: Arranging the results by gene group could make it easier to follow the results.
It might be helpful to show a PCA of the transcript data.
Re: PCA graphs of the transcriptome data were added: "Principal component analysis (PCA) of the transcriptome data showed similarity of the gene expression patterns in hybrids and Mimics (Figure S7)". Please see page 7, lines 4-5.

Detailed Comments
Q11: P4 L19-45: Instead of the detailed results, please outline the scientific questions guiding the research.

Re: Suggestion was followed, sentence was added in the manuscripts to outline the scientific questions: "To investigate whether Hybrid Mimics can be selected from other Arabidopsis hybrid combinations and to understand the molecular basis of increased plant size, we selected Hybrid Mimics from two hybrid systems involving other ecotypes of Arabidopsis". Please see page 4, lines 20-23.

Q12: P5 L3: Throughout the text, please define what you mean by similarity/similar.

Re: Suggestion was followed. Similarity was judged by statistic test. Text has been modified to description the term of comparison or “$P > 0.05$” was added after the statement.

For example:

- “Hybrids and Hybrid Mimic lines showed similarity in growth patterns as measured by increased rosette diameter compared to MPV (Figures 2a-b and S5a-b)”. Please see page 5, lines 10-11.
- “Four Hybrid Mimics (WL_HM2, 3, 4 and 5) had rosette diameter similar to the Ws/L*er* hybrids, and WL_HM1, 6, 7 had rosette sizes similar to the parent L*er* at 15 DAS (Figure S5c) ($P > 0.05$)”. Please see page 5, lines 23.
- “The remaining two HMs (CL_HM2 and 3) had rosette diameters similar to the parents at 15 DAS (Figure S5c) ($P > 0.05$)”. Page 6, line 20.
- “At 35 DAS, the intercross offspring had rosette diameters similar to the better parental Hybrid Mimic line WL_HM7 ($P > 0.05$)”. Page 14, line 13.
- “The offspring had plant sizes similar to the better parental Hybrid Mimic CL_HM4 at 15 DAS (P > 0.05)…”. Page 14, line 20.

Q13: P5 L17: ‘Small plant lines’ should be introduced/defined together with the hybrids, at least in Material and Methods.

Re: Suggestion was followed. Small plant lines were defined in the Material and Methods. “Selection for an F1-like phenotype or small plants was performed at 30 DAS based on rosette diameter and the time of flowering initiation…” Please see page 20, lines 16-18.

Q14: P5 L 29: Please show the growth rates. The rapid growth seems to take place earlier than 3 weeks after sowing.

Re: Data for the growth rates is provided in the Fig.S5. We agree with reviewer’ comments, description about growth rates have been corrected: “The hybrids and Mimics had rapid growth rates at approximately two to three weeks after sowing and and at later stages) (Figures 2a-b and S5a-b)”. Please see page 5, lines 23-24.

Q15: P5 L57: ‘high level of uniformity’: the hybrid mimics show higher variance than the other lines, e.g. in Fig. S6. How do you define ‘uniformity’?

Re: The uniformity is defined by the degree of the variation in samples of a statistical population. In our case it indicates the similarity of the plant size among individual plant within in one plant line as one giving population. We agreed with the reviewer’s comments regarding the uniformity of WL_HM5 and WL_HM7 is less than the F1 hybrids and other Hybrid Mimics. Correction has been made in the text. Please see page 6, lines 15-16.

Q16: P6 L29: The plants were analysed 25 DAS in this instance, at later time points in other cases. What is the rationale behind the changes?

Re: The rosette diameters of the plant sizes were measured at multiple time-points for growth pattern. 25 DAS was chosen as a time-point for transcriptome as: 1) all the Hybrid Mimics showed larger plant size than the parents. 2) Hybrids and most of the Hybrid Mimic lines have a higher growth rate than parents and small lines.

Q17: P6 L36-38: How was the cut-off chosen/defined? What about the fold-change? Were raw or adjusted P-values used – please indicate in Material and Methods? If raw P-values were used, how do the authors justify this?

Re: The choice of cut-off value was suggested by the bioinformatics analyst. Raw-p values generated by DEseq2 were used and no fold-change cut-off was applied for differentially expression genes. We did real-time PCR validation for a number of differentially expressed genes, the data from real-time PCR showed consistent results from the transcriptome data (Figure S9). These differentially expression genes were further justified by gene annotation and biological function. More detailed information was added in the material and methods and the reference for DEseq2 was added. Please see pages 22-23: “Transcriptome analysis”.

Q18: P6 L40: please give exact percentage.

Re: Percentage was given in the text.

Q19: P6 L43: ‘2053 genes’: please give % of expressed genes.

Re: Percentage was given in the text.

Q20: P9 L25: ‘activity’: unless enzyme activity was measured, this should be ‘expression’ or ‘transcription’.

Re: Correction has been made.

Q21: P9 L36-41: Please describe ‘line G’, and the corresponding experiment, as these 3 genes are not mentioned in the text of Wang et al. 2015.

Re: The descriptions of C24/Ler hybrids and Hybrid Mimic line G line were added in the text. “In crosses between the C24 and L*er* ecotypes, the F1 hybrids had substantial levels of hybrid vigor in vegetative biomass and plant size (Groszmann *et al.*, 2014). In the Hybrid Mimic line L2 (referred as HM-G here) selected from the C24/L*er* hybrid system (Wang *et al.*, 2015)….”. Please see page 10, lines 9-11.

Q22: P10 L5: How many are ‘some’ overlapping XTH genes?

Re: Correction has been made. “some” was replaced by “two to four *XTH* genes”.

Q23: P10 L21: C24/Ler hybrids were not introduced; see above P9 L36-41.

Re: The descriptions of C24/Ler hybrids and Hybrid Mimic line G line were added in the text. “In crosses between the C24 and L*er* ecotypes, the F1 hybrids had substantial levels of hybrid vigor in vegetative biomass and plant size (Groszmann *et al.*, 2014). In the Hybrid Mimic line

L2 (referred as HM-G here) selected from the C24/L*er* hybrid system (Wang *et al.*, 2015)….”. Please see page 10, lines 9-8.

Q24: P11 L18-30: Different PR genes are downregulated in the hybrid mimics, and also in a small plant line. This would suggest that there is no link with biomass in the WL cross.

Re: Our results indicate the down-regulation of defense response genes such as PR genes could contribute to increase plant size, and the up-regulation of defense response pathway genes are likely associated with the small plant size. This is not absolute as other pathways also impact the plant size. In the small plant line wl_sml1,

Q25: P11 L53: Are the senescence genes with changed expression involved in the defence response?

Re: Yes. There is some overlap regarding the genes in the senescence pathway and the defense response pathway.

Q26: P12 L3: ‘Plants with later flowering times have larger plant sizes’. This seems a contradiction to earlier results from the hybrid mimics (P5).

Re: Text has been modified: ‘Plants with later flowering times are more likely to have larger plant sizes due to a longer vegetative phase.’. Please see page 13, lines 4-5.

Q27: P13 L13: Please stress the point of crosses between 2 hybrids form the same parents (within system), or from different parents (between systems). Why were these particular lines chosen for the crosses?

Re: Firstly, for inter-crosses within system, two largest Hybrid Mimic lines are chosen based on rosette size and total fresh weight at 30 DAS. WL_HM4 and WL_HM7 were chosen in Ws/Ler system, CL_HM1 and 4 were chosen in Col/L*er* system. We made crosses CL_HM1x4, and WL_HM4x7.

Secondly, for Hybrid Mimic lines from different parents (between systems), crosses were made between the selected top 2 Hybrid Mimics from each system. We made crosses WL_HM7 x CL_HM4 and WL_HM4 x CL_HM1.

In the manuscript, we only presented the results from two crosses WL_HM4x7 and WL_HM7 x CL_HM4. Data from the other two crosses showing similar results was added in the text and supplementary material and figures, please see Figure S13.

Q28: P14 L8-12: This paragraph just states the results described earlier, without putting them in context or otherwise discussing them. Merging results and discussion could prevent this problem.

Re: Agree. This paragraph has been merged in to result section.

Q29: P14 L47: Please also discuss opposing views (e.g. Meyer et al. 2012, TPJ 71, 669-683), which detected no heterosis in Arabidopsis seeds.

Re: Discussion was added as reviewer's suggestion: ". In our experiments, the rates of seed germination were examined on Murashige and Skoog (MS) medium supplemented with 3% (wt/vol) sucrose using freshly collected seeds (approximately 4 weeks after seed collection). On moist soil, C24/Col hybrids had germination times similar to the faster germinating parent Col (48 hours after sowing), while parent C24 germinated approximately 20 hours later (Meyer *et al*., 2012). The different observations of the germination times of Arabidopsis hybrids compared to their parents can be due to differences of growth condition, the age of the seeds or different hybrid genotypes". Please see page 16, lines 4-11.

Q30: P16: Clock genes have also been associated with heterosis in Arabidopsis (e.g. Ni et al. 2009, Nature 457, 327-331). How does this fit with the presented results?

Re: We checked the gene expression of four clock genes:

- *CIRCADIAN CLOCK ASSOCIATED 1* (*CCA1*)/AT2G46830;
- *LATE ELONGATED HYPOCOTYL* (*LHY*)/AT1G01060;
- *TIMING OF CAB EXPRESSION 1* (*TOC1*)/AT5G61380;
- *GIGANTEA* (*GI*)/AT1G22770.

In our data it is not clear whether the expression of clock genes in the Hybrid, Mimics and small lines are associated with the plant sizes. For examples: *CCA1* and *LHY* were down-regulated in the Ws/Ler hybrids and six Mimic lines; *CCA1* and *LHY* are also downregulated in the two Ws/Ler small lines.

Q31: P18: Plant Material and growth conditions

Re: Reviewer's suggestions have been followed, questions have been answered. Missing information have been provided in the manuscript.

1. How were the parental seeds produced? Were there size differences between hybrid, hybrid Mimics and parental seeds?

Re: Seeds of parental lines, Hybrid Mimics and small plant lines were obtained through natural pollination without restricting the number of siliques unless specified. In Figure 9, the F1 hybrids (Ws/L*er* and Col/L*er*) and inter-cross offspring of Hybrid Mimics were produced by hand-pollinated; the silique-restricting procedure were applied for producing seeds of the control lines: Ws, L*er*, Col and parental Hybrids Mimics (Meyer *et al.*, 2004). We did not measure the seed sizes of hybrids, parents and Hybrid Mimics.

2. What means 'close to F1 hybrid range'?

Re: Unclear description "close to F1 hybrid range" has been removed.

3. How long were seeds kept in the dark at 4°C (stratification)?

Re: Information has been added in the Material and methods: "Seeds were kept at 4 °C for three days in the dark, then transferred into a growth room…".

4. Please describe the growth conditions for the soil-grown plants, including pot size and type of substrate.

Re: Information of growth condition, pot size and soil type have been provided: "At 15 days after sowing, each plate-grown seedling was transferred to a 65 mm W x 65 mm L x 100 mm H square pot containing soil (Debco Seed Raising & Superior Germinating Mix, Debco, Australia) and grown in the same growth room [16h light (22°C)/8h dark (18°C); light density: 120 -150 μmol photons $m^{-2}\cdot s^{-1}$]". Please see page 20, lines 5-8.

5. In Fig. 7 reciprocal hybrids are shown. Were they used throughout? Please clarify which type of hybrid was used in each experiment.

Re: Suggestions have been followed, in each figure hybrids have been labelled specifically. We do notice that in early growth stages there are growth differences between reciprocal hybrids, however there is no significant difference in rosette diameters of two reciprocal hybrids at 30 DAS under our growth condition, please see Figure S3.

Q32: P18-19: Recurrent selection…

1. Please define 'F1-like'.

Re: A sentence was added: "F1-like" is defined as: (1) plants showing IPV within the range of parents' IPV; (2) at 30 DAS plants had the largest rosette diameter in the selection population or at least had rosette dimeter similar to the rosette diameter of the F1 hybrids. The smallest plants were selected as the controls of large plants". Please see page 20, lines 19-22.

2. Please clarify the selection process. Did you select the biggest and smallest plants? Why did you not select equal numbers of plants in each category?

Re: Reviewer's suggests have been followed. The selection process was clarified: "Selection for an F1-like phenotype or small plants was performed at 30 DAS based on rosette diameter and the time of flowering initiation. Plants initiating flowering beyond the range of the IPV of parents were excluded from the selection. "F1-like" is defined as: (1) plants showing IPV within the range of parents' IPV; (2) at 30 DAS plants had the largest rosette diameter in the

selection population or at least had rosette dimeter similar to the rosette diameter of the F1 hybrids. The smallest plants were selected as the controls of large plants". Our main focus is to select Hybrid Mimic plants, so a small number of small lines were selected as negative controls.

3. L22: Did the plants fail after the initial selection? What is an 'unsatisfactory phenotype'?

Re: text has been changed to clarify the selection process: "Some F3 plant lines produced by large F2 plants were not selected due to their unsatisfactory phenotype of reduced plant sizes or flowering initiation time later than both parents in the F3 generation". Please see page 21, lines 8-11.

4. L3: please just list the 3 time points.

Re: reviewer's suggestion is followed.

Q33: P20: Transcriptome sample preparation…

1. L12: Ler is missing in the list of lines sampled?

Re: corrected.

2. L27-44: Unclear. How many actual replicates were used?

Re: Replicate information was added in the material and method: "Three biological replicates were collected per plant line. For the parents and hybrids, rosette leaves from three plants of each genotype were pooled as one biological replicate. For each Hybrid Mimic and small line, rosette leaves from one plant were collected as one biological replicate.". Please see page 22, lines 10-13.

Q34: P20-21: Transcriptome analysis

1. Please provide the pre-processing procedures and settings for the sequence alignment.

Re: Information was added in material and method: Alignment of sequenced reads was performed using STAR version 2.5.3a against the TAIR10 reference genome and and the araport11 annotation. The settings for the sequence alignment are:

--outFilterMismatchNmax 10 \

--outSAMtype BAM SortedByCoordinate \

--quantMode GeneCounts \

--outFilterMultimapNmax 10 \

--outSAMattrIHstart 0 \

--outSAMmapqUnique 255 \

--outSAMmultNmax -1 \

--chimSegmentMin 40

2. Please provide the script(s) and settings for the differential gene expression analysis.

The information of "The detailed scripts are available on request from the corresponding author' was added in the Material and methods.

3. Was the gene expression normalised to transcript length to avoid a potential bias towards long genes?

No, we used the raw counts of sequencing reads for each gene.

4. Which percentage of expressed genes was differentially expressed in the various hybrids and hybrid mimics?

Re: Percentages were added in the text.

5. L44: Please provide GEO accession no.

Re: GEO accession no. is provided.

Q35: P21: Gene ontology…

1. L40: 'Genes with reads ≥ 50 at least in one sample were considered expressed genes': this is not in agreement with L10-11, genes with minimum reads ≥ 50 across all the samples'.

Re: Correction has been made.

2. L45: Why were not adjusted P-values used?

Re: Go annotation was redone. Significant Go term is defined when significant level < 0.05 (Statistical test method: Fisher, Multi_test adjustment method: Hocheberg FDR). Minimum number of mapping entries = 5.

Q36: Figure 1

1. Are the values for F1 hybrids means of reciprocal hybrids, or was just one hybrid selected (Ws female, Ler male), and if so, why?

Re: Only one hybrid was used for each combination for the data in Fig.1 (ColxL*er*, WsxL*er*). There is no significant difference in rosette diameters of two reciprocal hybrids at 30 DAS under our growth condition, please see Figure S3 as an example.

2. Please add MPV for fresh weight

Re: Dotted lines have been added as an indicator of MPV.

3. Please discuss the incongruent relation between rosette diameter and fresh weight – why do lines with smaller rosette diameter have larger fresh weight?

Re: As reviewer's suggestion, for each plant line, fresh weights of rosette leaves and shoots were represented separately to avoid confusion.

4. Please discuss differences in rosette/shoot ratios in more detail.

Re: Reviewer's suggestion has been followed, please see page 6: "At 30 DAS, plants are at different developmental stages due to the flowering time differences. The ratios between the biomass of rosette leaves and shoots were different in the parents, hybrids and F7 lines……The overall fresh weights of WL_HM lines were less than the Ws/L*er* hybrid due to a less well developed shoot at 30 DAS, since the WL_HMs had delayed flowering initiation than the Ws/L*er* F1 hybrids".

Q37: Figure 2

1. Please also show growth rates.

Growth rates of each plant lines have been provided in Figure S5.

2. Why show smoothed lines with only 3 data points?

Graph has been changed to scatter with straight lines.

Q38: Figure 3: Please define 'similar to F1', 'same trend' and 'opposite to F1'.

Re: Please see figure legends.

Q39: Figure 6: Please explain the differences between the hybrid mimics in relation to your theory.

Re: Please see discussion section, page17- 18: "The down-regulation of defense pathway genes was observed in all Ws/L*er* Hybrid Mimics and one hybrid Mimic from the Col/L*er* system (CL_HM1), but not in CL_HM2, 3 and 4. Both small lines selected from the Col/L*er*

system had defense genes up-regulated with all five PR genes expressed at least two-fold higher than the MPV”.

Q40: Table 1:

1. Please indicate number of replicates.

Re: Information is added in the Material and method: “At least 10 seeds per line were scored as one replicate. The data represent the average value from two to four replicates”.

2. The red type indicates the time point when the majority of seed from a line (>80%) have germinated.

Re: corrected.

Q41: Fig. S6: Hybrid mimics display a large variance (especially in panel a), therefore while their mean rosette diameter is indeed larger, they are not more uniform!

Agree. Text has been corrected regarding the uniformity of the WL_HM5 and HM6.

Q42: Fig. S7: Filter 1 – are only the significant DEGs involved?

Yes.

Q43: Fig. S8: This experiment should be described as the others, even if the lines are from an earlier publication.

Re: Reviewer’s suggestion has been followed. The HM-G was introduced and described in the text.

Q44: Minor Comments: All agreed and changed.

P2 L22: The two hybrids showing different levels of heterosis. (show)

P5 L47: Remove additional ‘to’.

P8 L 52 UDP-D-APIOSE/UDP-D-XYLOSE SYNTHASE 1 (AXS1) involved in the cell wall: add ‘is’ after (AXS1)

P12 L 57: should be ‘cell wall related genes’

P13 L3: In the Ws/Ler Hybrid Mimic plants defense pathway genes (plant)
